# Mitophagy in cisplatin-induced kidney injury: regulatory mechanisms and therapeutic targets

**DOI:** 10.1080/07853890.2026.2672141

**Published:** 2026-05-27

**Authors:** Cheng Yuan, Jinzhou Jiang, Jinxing Ji, Lihua Ni

**Affiliations:** a Department of Oncology, Yichang Central People’s Hospital and The First College of Clinical Medical Science, China Three Gorges University Yichang, Hubei, China; b Tumor Prevention and Treatment Center, Three Gorges University and Cancer Research Institute of Three Gorges University Yichang, Hubei, China; c Clinical Medical Research Center for Precision Diagnosis and Treatment of Lung Cancer and Management of Advanced Cancer Pain of Hubei Province; d Central Laboratory, The First College of Clinical Medical Science, China Three Gorges University & Yichang Central People’s Hospital, Yichang, Hubei, China; e Key Laboratory of Tumor Microenvironment and Immunotherapy of Hubei Province, Yichang, Hubei, China; f Department of Nephrology, Zhongnan Hospital of Wuhan University, Wuhan, Hubei, China

**Keywords:** Mitophagy, cisplatin-induced kidney injury, therapeutic targets, mtROS, PINK1/PARKIN pathway, programmed cell death

## Abstract

**Background:**

Cisplatin is a first-line chemotherapeutic agent widely used in clinical oncology, but its clinical utility is severely limited by cisplatin-induced acute kidney injury (Cis-AKI). Renal tubular epithelial cells (RTECs) are the main target of cisplatin-induced damage, and the pathogenesis involves oxidative stress, inflammatory response, and multiple types of programmed cell death. As a core mitochondrial quality control mechanism, mitophagy is closely related to the above pathological processes, but its overall regulatory network in Cis-AKI remains to be systematically clarified.

**Main body:**

This review systematically summarizes the core pathological mechanisms of cisplatin-induced nephrotoxicity, including mitochondrial dysfunction, oxidative stress, inflammation, apoptosis, pyroptosis, and ferroptosis. It focuses on the molecular pathways of mitophagy (PINK1/PARKIN-dependent and -independent pathways), the bidirectional crosstalk between mitophagy and the aforementioned pathological events, and the dual role of mitophagy in Cis-AKI. In addition, this review collates preclinical progress of mitophagy regulators and their renoprotective effects, and analyzes the current obstacles to clinical translation.

**Conclusion:**

Mitophagy serves as a key regulatory node in Cis-AKI and can simultaneously ameliorate multiple injury pathways by clearing damaged mitochondria and reducing mtROS. Moderate mitophagy plays a renoprotective role, while excessive mitophagy aggravates renal injury. Targeted and precise regulation of mitophagy is expected to become a new strategy for the prevention and treatment of cisplatin-induced nephrotoxicity.

## 1. Introduction

Cancer has become a prominent global cause of mortality, with its prevalence steadily increasing [1,2]. Chemotherapy serves as a pivotal component in the treatment of tumors, significantly enhancing patient survival rates [3–5]. Nevertheless, the occurrence of drug-induced nephrotoxicity, notably Cis-AKI, poses a significant clinical obstacle [6]. These injuries can advance to acute kidney injury (AKI) or chronic kidney disease (CKD), profoundly impacting patients’ well-being and constraining its clinical utility [7]. Despite extensive investigations, the precise molecular mechanisms underlying chemotherapy-triggered nephrotoxicity remain incompletely understood, leading to a dearth of targeted preventive and therapeutic approaches.

Prior studies have established that chemotherapeutic agents (e.g. cisplatin, cyclophosphamide, tacrolimus) induce kidney injury *via* a network of interconnected mechanisms, including inflammation, oxidative stress, and three major types of programmed cell death (PCD)-apoptosis, pyroptosis, and ferroptosis-alongside autophagic dysregulation [8,9]^.^ Among these agents, cisplatin (a frontline platinum-based drug) is the most well-documented for its nephrotoxicity, which primarily arises from direct mitochondrial damage: it disrupts mitochondrial membrane potential, increases mitochondrial reactive oxygen species (mtROS) production, and triggers inflammatory responses, ultimately impairing renal function [10–12]^.^ Given its pivotal role in cellular energy metabolism and the regulation of PCD, mitochondrial dysfunction plays a crucial role in the development of kidney injury [13]. Notably, mitophagy is the only mechanism to selectively clear damaged mitochondria, making it a potential ‘unified regulatory node’ for mitigating multiple pathogenic pathways in Cis-AKI.

Since its proposal by Christian de Duve in 1963, the concept of autophagy has given rise to various selective forms targeting distinct organelles, such as mitophagy and reticulophagy [14,15]. Mitophagy, in particular, serves as a specialized defense mechanism against oxidative stress and mitochondrial dysfunction, exhibiting a complex regulatory framework [16]. This regulation encompasses not only the PINK1/PARKIN signaling axis and autophagy-related proteins (e.g. Microtubule-Associated Protein 1 Light Chain 3, LC3) – a core marker for monitoring autophagic activity, including mitophagy – but also other protein-mediated pathways that directly govern mitophagy [17–19]. In response to the damage incurred by RTECs following chemotherapy, cells induce mitophagy to eliminate dysfunctional mitochondria and uphold cellular homeostasis [20]. While moderate mitophagy functions protectively by eliminating impaired mitochondria, excessive activation (characterized by an increase in LC3-II/LC3-I ratio and a reduction in mitochondrial mass) can induce cell death and exacerbate cellular injury, representing a typical context-dependent bidirectional regulatory effect in pathological processes [21,22]. These intricate molecular interactions are intricately linked to kidney injury repair. A thorough exploration of their regulatory mechanisms holds significant implications for the advancement of innovative renal protection strategies.

This review systematically examines the molecular mechanisms underlying Cis-AKI, emphasizing the regulatory role of mitophagy and its interactions with inflammation, oxidative stress, and PCD pathways, with mtROS serving as a central hub linking mitochondrial dysfunction to these interconnected pathological processes. By assessing recent advancements in mitophagy regulators in preclinical research, our goal is to establish a theoretical framework for developing precise intervention strategies that can preserve chemotherapy efficacy while mitigating renal damage. The subsequent sections will analyze: 1) The pathogenesis of Cis-AKI; 2) The interplay between mitophagy and PCD pathways; 3) The therapeutic implications of modulating mitophagy in Cis-AKI.

## 2. Pathological mechanisms of Cis-AKI

Renal injury is a prevalent and serious complication associated with chemotherapeutic agents used in clinical oncology. The pathogenesis of this condition is marked by the intricate interplay of multiple factors and pathways. Prior research has validated the intricate nature of this injury, which encompasses diverse pathophysiological mechanisms including oxidative stress, inflammation, PCD, and mitochondrial dysfunction. Importantly, these mechanisms are not independent entities but rather intricately interconnected through a network of signaling molecules, culminating in renal impairment.

Oxidative stress plays a pivotal role as an early driver in kidney injury, linking drug toxicity, cellular damage, and inflammation escalation. Chemotherapeutic agents like cisplatin disrupt renal redox balance through various pathways. Studies in animal models have revealed that cisplatin administration leads to impaired fatty acid oxidation and extensive lipid accumulation in kidney cells, resulting in increased mtROS production, apoptosis, and kidney injury [23]. Moreover, RTECs experience elevated mtROS levels, heightened lipid peroxidation, reduced glutathione levels, and diminished tissue antioxidant capacity, which intensifies oxidative stress [24]. This detrimental cycle ultimately compromises the antioxidant defense system. Despite a positive correlation between mtROS levels and kidney injury severity, antioxidant use does not prevent cell death, suggesting that mtROS are not the primary cause of cell death [25]. It is proposed that mtROS may primarily exacerbate damage by activating downstream inflammatory and apoptotic pathways, a notion supported by subsequent discussions.

The inflammatory response plays a central role in kidney injury and shows high tissue specificity. It acts as an early driver of kidney injury and is closely associated with injury progression and fibrosis. Cisplatin can trigger inflammatory signaling through multiple mechanisms, and its effects are primarily on RTECs. The specific mechanisms are described below: Cisplatin can activate signal transducer and activator of transcription 1 (STAT1), which in turn upregulates high mobility group box 1 (HMGB1); this process further promotes the activation of Nuclear Factor-kappa B (NF-κB) and the production of inflammatory factors. Ultimately, it exacerbates tubular injury and renal fibrosis, leading to kidney damage [26,27]. Cisplatin can also directly increase the levels of inflammatory factors in the kidney. These include tumor necrosis factor-α (TNF-α), monocyte chemoattractant protein 1 (MCP-1), and intercellular adhesion molecule 1 (ICAM-1). Cisplatin promotes the degradation of inhibitor κB (IκB), enhances NF-κB activity, and induces kidney injury by activating the inflammatory pathway [28]. Similarly, in the presence of cisplatin, the chemokine C-X-C motif chemokine ligand 1(CXCL1) binds to its receptor C-X-C motif chemokine receptor 2(CXCR2). This binding not only recruits neutrophils but also further amplifies NF-κB signaling through the phosphorylation of p38 mitogen-activated protein kinase (MAPK). This forms a self-amplifying inflammatory loop [29]. Activation of this NF-κB-dependent inflammatory pathway leads to a marked increase in cytokines such as IL-1β, TNF-α, MCP-1, and caspase-1. These changes correlate with the severity of kidney injury [30]. These findings provide experimental evidence supporting the pivotal role of the inflammatory response in kidney injury.

PCD comprises various pathways, including apoptosis, ferroptosis, and pyroptosis [31]. Recent research has highlighted the close relationship between PCD and kidney injury following chemotherapy. These pathways govern cell death *via* distinct molecular mechanisms, thereby impacting the equilibrium and functionality of renal tissues. Apoptosis, as the principal form of PCD, can be triggered in RTECs by chemotherapeutic agents like cisplatin. Cisplatin initiates both the intrinsic mitochondrial pathway and the extrinsic death receptor pathway, culminating in renal dysfunction. Apoptosis in Cis-AKI displays hallmark mitochondrial-dependent features. Cisplatin disrupts the equilibrium of Bcl-2 family proteins, heightening outer mitochondrial membrane (OMM) permeability, leading to the substantial release of cytochrome C (Cyt C) into the cytoplasm, and activating the caspase cascade to induce apoptosis in RTECs [32]. It is accompanied by activation of NF-κB and NOD-like receptor protein 3(NLRP3) inflammasome, increased expression of apoptosis-related markers, and induction of RTECs apoptosis [33]. Moreover, cisplatin can induce apoptosis in RTECs by triggering the Fas ligand-mediated extrinsic death receptor pathway [34]. Studies have also demonstrated that cisplatin-induced oxidative stress can concomitantly activate the ferroptosis pathway, characterized by glutathione depletion, Glutathione Peroxidase 4 (GPX4) activity inhibition, and lipid peroxide accumulation, collectively causing cell damage alongside apoptosis [35]. The activation of this dual death pathway may underlie the challenge in completely mitigating cisplatin nephrotoxicity. Additionally, pyroptosis, an alternative PCD mechanism, is initially characterized as a caspase1- and inflammasome-dependent pathway that releases inflammatory mediators by forming cell membrane pores mediated by Gasdermin (GSDM) proteins. It is now recognized that the GSDM protein family can also be activated through inflammasome- and caspase-independent routes, exacerbating the renal inflammatory response and interplaying with other cell death pathways [36].

Platinum-based drugs induce DNA damage as a primary toxic mechanism. Upon cellular uptake, these drugs undergo hydration to generate positively charged platinum species that can react with nucleophilic sites on DNA, leading to the formation of DNA cross-links and subsequent damage [37]. The resulting cisplatin-DNA complexes impede normal DNA replication and transcription, ultimately triggering apoptosis [38]. Recent spatiotemporal transcriptomics studies have revealed that cisplatin-induced DNA damage predominantly occurs early on in the cortical proximal tubules (CPT) and outer medullary proximal tubules (OMPT) rather than in the glomeruli (GM). Sustained DNA damage response is most pronounced in the OMPT, indicating that Cis-AKI primarily affects this region [39]. These findings underscore significant site-specific disparities in chemotherapeutic drug-induced kidney injury, highlighting the importance of further investigating the spatial distribution characteristics in this context.

Mitochondrial dysfunction is identified as the ultimate common pathway in the development of Cis-AKI. Research indicates that following methotrexate (MTX) administration, various mitochondrial parameters such as mitochondrial membrane potential, mitochondrial dehydrogenase activity, mitochondrial glutathione levels, and ATP content decrease, while lipid peroxidation and mitochondrial permeabilization increase [24]. Pharmacological interventions have been shown to ameliorate mitochondrial dysfunction, restore renal tubule and mitochondrial morphology, and consequently protect against Cis-AKI [40]. Several studies have demonstrated that the mitigation of impaired mitochondria through mitophagy, facilitated by multiple pathways, plays a crucial role in restoring cellular or organ function [41,42]. These findings underscore the significance of enhancing mitochondrial function in alleviating renal injury.

Autophagy, specifically mitophagy, plays a dual role in Cis-AKI, a focal point of this study. Mitophagy is pivotal in PCD, cell metabolism, and survival. While moderate mitophagy can be protective by eliminating damaged mitochondria, excessive mitophagy can trigger cell death [43]. For example, in a Cis-AKI model, mitophagy-deficient (PARKIN-knockout) mice exhibit more severe mitochondrial damage and renal tubular injury [41], underscoring the protective role of moderate mitophagy in Cis-AKI. Conversely, exposure to cadmium and polystyrene nanoplastics (PSNP) has shown that oxidative stress, ferroptosis, and excessive mitophagy act synergistically to worsen kidney injury, highlighting a complex interplay between mitophagy and cellular damage [22]. An investigation using an organoid model of tacrolimus nephrotoxicity revealed that tacrolimus treatment induces oxidative stress, mitochondrial dysfunction, and heightened autophagic activity, elucidating a portion of its nephrotoxic mechanism [11]. Furthermore, in systemic diseases like F- box and Leucine Rich Repeat Protein 4 (FBXL4)-mutated mitochondrial DNA (mtDNA) depletion syndrome, excessive BNIP3/BNIP3L-dependent mitophagy disrupts mitochondrial homeostasis [44]-a phenomenon similar to the ‘excessive mitophagy exacerbating cell death’ observed in Cis-AKI. Additionally, in a cerebellar degeneration model, mitochondrial transplantation mitigated mitophagy and delayed cell death [45], suggesting that targeting mitophagy intensity (not just presence) may be a universal protective strategy, including in Cis-AKI.

**Figure 1.**
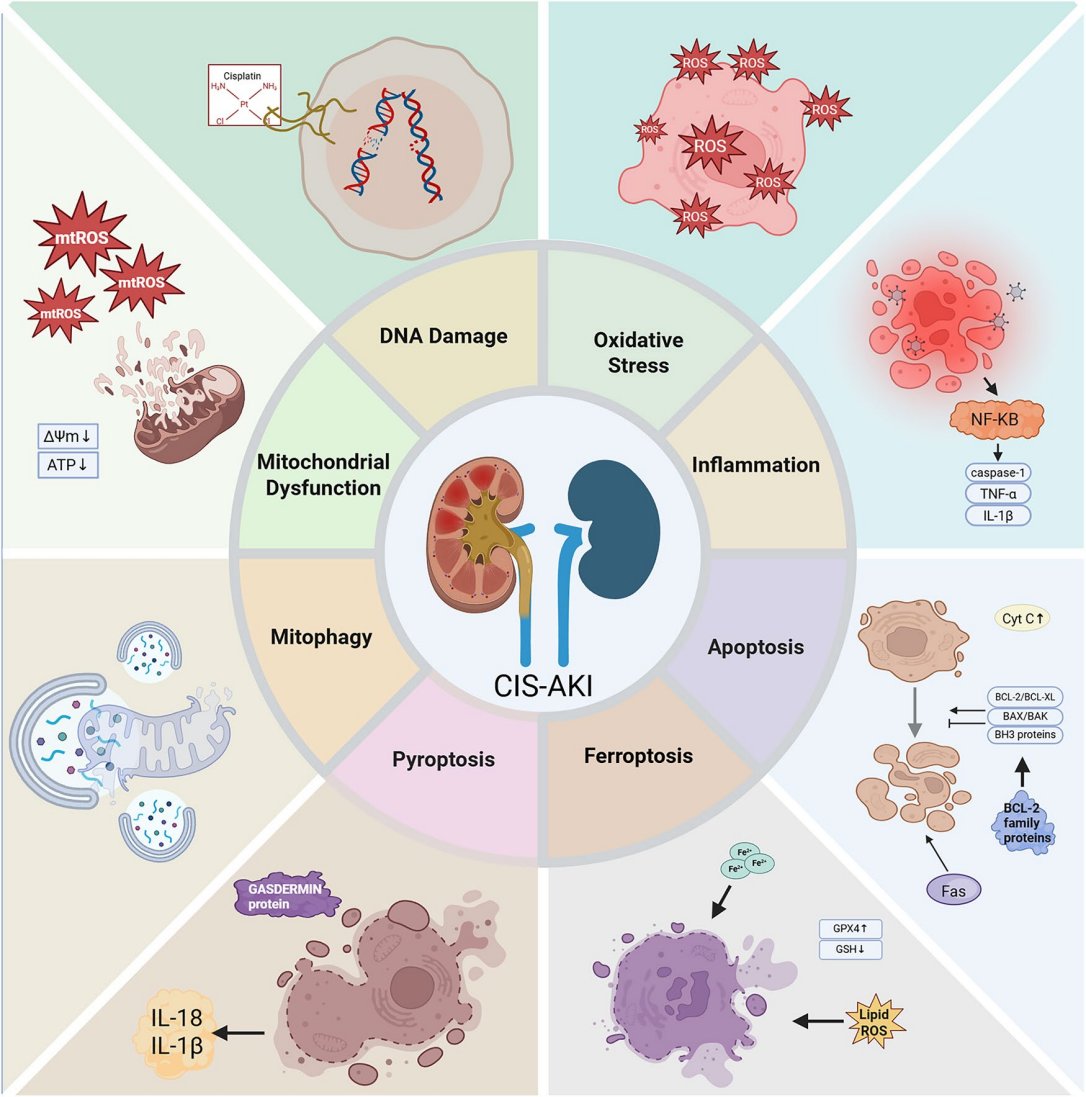
Mechanism network of Cis-AKI. The multi-mechanism network of Cis-AKI, including DNA damage, oxidative stress, inflammation, apoptosis, ferroptosis, pyroptosis, mitochondrial dysfunction, and mitophagy. Note: **mtROS**: Mitochondrial reactive oxygen species; **Lipid ROS**: Lipid reactive oxygen species; **ROS**: Reactive oxygen species.

Cis-AKI is a multifaceted pathological phenomenon driven by the concerted interplay of multiple mechanisms. Rather than operating independently, these mechanisms intricately intertwine through the convergence of diverse signaling pathways, culminating in cellular dysfunction. The direct associations between Cis-AKI and these factors are shown in Figure 1. This study seeks to elucidate the interplay between mitophagy and apoptosis and their implications for Cis-AKI. A comprehensive comprehension of these interactions, particularly the central involvement of mitophagy in each pathway, will furnish a crucial theoretical foundation for devising renal safeguarding interventions. Given that mitochondrial dysfunction is the core driver of Cis-AKI, understanding the biological functions of mitochondria and the regulatory network of mitophagy (the only mechanism to clear damaged mitochondria) is essential for elucidating targeted intervention strategies, which will be discussed in the following sections.

## 3. Mitochondrial homeostasis and the molecular regulation of mitophagy

### 3.1. Mitochondrial function

Mitochondria are the cellular energy-producing sites and metabolic regulatory hubs, playing a vital role in various organs. They produce ATP to fuel cellular functions through oxidative phosphorylation (OXPHOS) and possess a distinct genome [46]. Furthermore, mitochondria participate in essential physiological processes including amino acid and nucleotide synthesis, iron-sulfur cluster biosynthesis, intermediary metabolism, and regulation of calcium ion homeostasis [47]. Maintenance of mitochondrial morphology, quantity, and function is achieved through ongoing fusion (mediated by Mitofusin 1 (MFN1), Mitofusin 2 (MFN2), and optic atrophy 1 (OPA1) proteins) and fission (regulated by Dynamin-Related Protein 1 (DRP1) and Fission 1 (FIS1) proteins), both processes are dependent on the mitochondrial membrane potential and lipid microenvironment. This dynamic equilibrium is critical for mitochondrial repair [48]. As a damage sensor, PINK1 senses the integrity of the mitochondrial import pathway and activates PARKIN when import is impaired. As an effector, PARKIN labels damaged mitochondria *via* ubiquitination to provide a targeting basis for mitophagy and other mitochondrial quality control processes. Together, they synergistically mediate mitophagy [49,50].

Mitochondria play crucial roles in maintaining homeostasis in various tissues and organs. In the heart, mitochondria not only support cardiomyocytes with energy production through oxidative phosphorylation but also regulate excitation-contraction coupling by participating in calcium ion cycling.Specifically, mitochondrial dysfunction triggers increased generation of mtROS, resulting in oxidative stress that activates mitochondrial fission, fusion, and mitophagy processes. These reactions are closely linked to cardiovascular diseases such as myocardial hypertrophy and heart failure [51]. Similarly, in skeletal muscle, mitochondria are essential for energy supply. Research indicates that mitochondrial dysfunction due to PARKIN gene knockout in a Parkinson’s disease (PD) model can lead to muscle atrophy [52]. Moreover, decreased mitochondrial function accelerates sarcopenia progression, underscoring the critical role of mitochondrial homeostasis in skeletal muscle development [53]. In the liver, mitochondria primarily facilitate fatty acid β-oxidation and ketone body production, contributing to the integrated regulation of glucose, lipid, and amino acid metabolism [54]. Aberrant mitochondrial function in non-alcoholic fatty liver disease (NAFLD) results in lipid accumulation, triggering hepatic cell fibrosis and inflammatory responses. Restoring mitophagy may reverse this pathological process [55]. Research on liver cancer has demonstrated that modulating mitophagy can impede tumor growth by regulating mitochondrial function, emphasizing the pivotal role of mitochondria in liver diseases [56]. The kidney, as an organ with high energy demands, regulates water and electrolytes and heavily relies on mitochondrial energy supply [57]. Mitochondrial dysfunction is closely associated with AKI and CKD, leading to cell death, tissue injury, and potential organ failure [58]. For instance, in iodine contrast-induced kidney injury, mitochondrial damage induces an mtROS burst, causing oxidative stress and apoptosis, resulting in RTECs damage [59]. In brain tissue, mitochondria fuel neurons and glial cells, supporting synaptic plasticity. Studies have shown that mitochondrial dysfunction manifests early in Alzheimer’s disease (AD) patients, linked to disrupted mitochondrial dynamic renewal and autophagy [60–62]. Mutations in PARKIN and PINK1 genes can induce abnormal mitochondrial function, contributing to early-onset PD [63,64].

The aforementioned studies unequivocally establish the pivotal involvement of mitochondria in the pathogenesis and progression of diverse organ pathologies, owing to their multifunctionality and intricate regulation. Subsequent discussion will delve into elucidating the molecular underpinnings of mitophagy in nephrotoxicity induced by chemotherapeutic agents, offering novel insights for future investigations.

### 3.2. Molecular pathways and regulatory networks of mitophagy

As previously mentioned, autophagy encompasses various types, including mitophagy, reticulophagy, golgiophagy, nucleophagy, and ribophagy, each characterized by distinct action targets and mechanisms [15]. Mitophagy, unlike non-selective autophagy (which degrades cytoplasmic components broadly), is a tightly regulated process that selectively identifies and removes damaged, depolarized, or dysfunctional mitochondria. This selective process is crucial for preserving mitochondrial network homeostasis [43].

The current understanding of mitophagy primarily relies on the investigation of the PINK1/PARKIN signaling pathway, initially identified in Parkinson’s disease research. Mutations in PINK1 or PARKIN (key genes in this pathway) are closely linked to autosomal recessive early-onset Parkinson’s disease [63]. PINK1 serves as a key mitochondrial protein and a central sensor for maintaining mitochondrial quality. Upon loss of mitochondrial membrane potential, PINK1 accumulates on impaired mitochondria, is stabilized on the OMM, interacts with the Translocase of the Outer Mitochondrial Membrane (TOM) complex, and is activated through autophosphorylation. Activation is regulated by both phosphorylation and oxidative modification [65]. PINK1 phosphorylates Ser65 of ubiquitin to generate pUb, signaling the recruitment of PARKIN. Initially inhibited, PARKIN undergoes a conformational change upon binding to pUb, releasing the Ubl domain. Subsequently, PINK1 phosphorylates Ser65 of PARKIN’s Ubl domain, fully activating PARKIN and initiating mitophagy [66]. Lack of PINK1 exacerbates mitochondrial dysfunction, impairing mitochondrial function and increasing sensitivity to oxidative stress [67]. PARKIN, an E3 ubiquitin ligase, directly ubiquitinates substrate proteins, promoting their degradation *via* the proteasome pathway. It collaborates with PINK1 to create ubiquitin chains on the OMM. PINK1 phosphorylates ubiquitin to tag damaged mitochondria, recruiting autophagy receptors for mitophagy initiation, while PARKIN amplifies the signal through ubiquitination, expediting mitophagy [68,69]. Accumulating evidence highlights the significance of PINK1’s localization and accumulation on the OMM, along with its specific recruitment and activation of PARKIN, as the fundamental mechanism of this pathway. PINK1 can phosphorylate MFN2 to form a PINK1-MFN2-PARKIN complex crucial for targeted removal of damaged mitochondria [70]. The PINK1/PARKIN pathway plays a pivotal role in regulating mitochondrial quality control and the autophagic process.

Aberrations in PINK1/PARKIN-dependent mitophagy are intricately linked to various diseases. Originally identified in Parkinson’s disease research, this pathway has now been implicated in multiple pathological processes [64]. In dystrophic cardiomyopathy, diminished expression of proteins associated with PINK1/PARKIN-dependent mitophagy indicates a pivotal role of mitophagy defects in disease pathogenesis [71]. Studies in animal models of liver fibrosis demonstrate that enhancing mitophagy can ameliorate the accumulation of dysfunctional mitochondria and alleviate liver fibrosis [72]. Moderate activation of mitophagy has been shown to restore exhausted β cell function in type 2 diabetes [73]. Conversely, in contrast-induced nephropathy, inhibition of mitophagy through gene silencing exacerbates mitochondrial damage and apoptosis, underscoring the central role of this pathway in mitophagy regulation [59]. Furthermore, dysregulation of the PINK1/PARKIN pathway is widespread, from tumors like lung cancer to acute injuries such as ischemia-reperfusion injury and sepsis [74–77], underscoring the broad pathophysiological relevance of PINK1/PARKIN pathway-mediated mitophagy.

The regulation of this pathway involves multiple levels: transcriptionally, the tumor suppressor p53 inhibits mitophagy by suppressing the PINK1 promoter and reducing its mRNA and protein expression [78], thereby inhibiting PINK1/PARKIN-dependent mitophagy. This negative feedback may prevent excessive mitophagy during early DNA damage repair, balancing cell survival and organelle clearance; post-translationally, reduced mitochondrial processing peptidase (MPP) levels lead to PINK1 accumulation on mitochondria, enhancing PARKIN recruitment and autophagic activity [79]. Additionally, Ubiquitin(Phospho-Ser65) facilitates PINK1-mediated PARKIN phosphorylation at Ser65, maximizing PARKIN E3 ligase activity [80]. USP8, a deubiquitinase, disrupts PARKIN-dependent ubiquitin chains, modulating mitophagy [81]. Other deubiquitinases like USP30 and USP33 also regulate mitophagy similarly [82–84]. These findings underscore deubiquitination as a crucial mechanism in mitophagy regulation.

In addition to the canonical PINK1/PARKIN-dependent pathway, alternative mechanisms exist for activating mitophagy that operate independently of this pathway.

LC3 is a key signature protein that remains present throughout all stages of autophagosome formation during autophagy, and its lipidated form (LC3-II) specifically localizes to autophagosomal membranes – an attribute that makes it the most widely used marker for tracking autophagosome dynamics. Moreover, its post-translational modifications, subcellular localization changes, and protein-protein interactions directly determine the initiation, progression, and functional execution of autophagic flux; it is these properties that collectively secure its irreplaceable core position in the molecular regulatory network of autophagy [85,86]^.^ As a specific marker for autophagosomes in mammals, LC3-II has been widely used to investigate autophagic processes in neurodegenerative and neuromuscular disorders, tumorigenesis, as well as bacterial and viral infections [87,88]. Currently, LC3-II is one of the most commonly used and core molecular markers in autophagy and mitophagy research, and its dynamic changes are often combined with other markers (e.g. p62/SQSTM1) to evaluate autophagic and mitophagic flux [89,90]

Under specific conditions, multiple OMM receptors regulate mitophagy by interacting directly or indirectly with core autophagy proteins. These receptors are activated during hypoxia or stress and facilitate the removal of damaged mitochondria by interacting with LC3-II. For instance, Activating Molecule in Beclin-1-Regulated Autophagy (AMBRA1) not only interacts with PARKIN to enhance its stability and increase ubiquitination, thereby promoting PINK1/PARKIN-dependent mitophagy, but also independently initiates mitophagy by binding to the autophagosome adaptor protein LC3-II *via* the LC3-interacting region (LIR) [91]. Another example is FUN14 domain-containing protein 1 (FUNDC1), an OMM-localized mitophagy receptor that initiates mitophagy *via* binding to LC3-II under hypoxia [92]. FUNDC1 recruits Unc-51 Like Autophagy Activating Kinase 1 (ULK1) to damaged mitochondria, where ULK1 phosphorylates FUNDC1 to further promote mitophagy [93]. Another key receptor is BCL2/adenovirus E1B 19 kDa protein-interacting protein 3-like (commonly abbreviated as BNIP3L or NIX), which directly binds to LC3-II to mediate mitophagy and plays a role in mitochondrial clearance during erythrocyte maturation. Phosphorylation of NIX enhances this process [94,95]. Moreover, the inner mitochondrial membrane (IMM) protein prohibitin 2 (PHB2) participates in eukaryotic mitophagy by interacting with LC3-II [96]. Additionally, FK506 binding protein 8 (FKBP8) induces mitophagy by specifically recruiting LC3-II [97].

Recent research has revealed that the Ras-related protein Rab-9 (Rab9)-dependent alternative mitophagy pathway is involved in the regulation of mitochondrial function. A protein complex consisting of ULK1, Rab9, Receptor-interacting serine/threonine-protein kinase 1(Rip1), and DRP1 triggers mitophagy by phosphorylating Rab9 (at Ser179 *via* ULK1) and DRP1 (at Ser616 *via* Rip1), independent of LC3-II [98]. ULK1 phosphorylated at Ser555 activates the alternative mitophagy pathway by binding to Rab9, which confers cardioprotective effects [99,100]. DRP1 is known to mediate conventional mitophagy through mitochondrial fission, and it can also participate in alternative mitophagy by interacting with Rab9 following phosphorylation at Ser616 [101,102].

The receptor-mediated pathways and associated regulatory mechanisms confer diversity and intricacy to mitophagy, presenting challenges to the study of this process. In addition to the canonical and alternative pathways of mitophagy, several auxiliary factors indirectly regulate mitophagy by modulating mitochondrial dynamics or signal transduction, and their tissue-specific roles further highlight the complexity of mitophagy regulation.

### 3.3. Auxiliary regulatory factors of mitophagy

Apart from receptors directly involved in mitophagy, certain factors indirectly influence the autophagic process by modulating mitochondrial dynamics,or signal transduction. A significant player in this regard is DRP1, which plays a crucial role in mitochondrial fission [101]. However, DRP1 exerts tissue-specific functions: in cardiomyocytes, DRP1 deficiency impairs mitophagy by blocking mitochondrial fragmentation, leading to cardiac dysfunction [103]; in contrast, in ischemic nephropathy models, reducing DRP1 levels protects RTECs by preventing excessive mitochondrial fission (which would otherwise release mtROS), thereby preserving mitochondrial integrity [104]. This tissue-specific dual effect highlights that DRP1 modulates mitochondrial homeostasis *via* distinct mechanisms depending on the organ microenvironment.

Multiple regulators exhibit multifunctionality in PINK1/PARKIN-dependent and independent mitophagy pathways, which is mainly reflected in the following aspects.

For example, in PINK1/PARKIN-dependent mitophagy, pro-survival members of the Bcl-2 family antagonize PINK1/PARKIN-dependent mitophagy in a Beclin-1/BAX/BAK-independent manner. They directly bind to the PINK1/PARKIN complex and inhibit PARKIN translocation to depolarized mitochondria. Other members of this family, such as BH3-only proteins or their mimetics, can neutralize this inhibitory effect and positively promote mitophagy. Considering their roles in regulating apoptosis and mitochondrial fission/fusion, Bcl-2 family proteins are regarded as global regulators of mitochondrial homeostasis [105]. P62/SQSTM1 and voltage-dependent anion channel protein 1 (VDAC1) act as connecting molecules in this pathway and play important roles. Both p62/SQSTM1 and VDAC1 can mediate mitochondrial aggregation and recruitment, but neither is essential for mitophagy. This indicates that the core executive mechanisms of mitochondrial aggregation and mitophagy are independent at the molecular level, reflecting the modular, separable and highly redundant characteristics of the mitophagy pathway [106,107]. SMAD3 can positively activate PINK1 transcription by binding to the PINK1 promoter. Meanwhile, PINK1 can in turn directly phosphorylate SMAD3 at the Ser423/425 sites to enhance its transcriptional activity, forming a positive feedback loop that synergistically promotes mitophagy [108]. RBX2-CRL5, a ubiquitin ligase localized on the outer mitochondrial membrane, enhances mitophagy by directly mediating the ubiquitination of outer mitochondrial membrane proteins and stabilizing PINK1 protein. This regulatory process is independent of PARKIN [109]. TMX2 combines with VDAC2/3 to promote PARKIN recruitment to dysfunctional mitochondria, thereby enhancing protective mitophagy. Oxidative stress-induced upregulation of TMX2 expression can strengthen this process [110]. In addition, TAU, SAM50, and others have also been identified as negative regulators of PINK1/PARKIN-dependent mitophagy [111,112].

Of course, other regulatory factors are also involved in PINK1/PARKIN-independent mitophagy. As a key inflammatory activator, NF-κB can activate the NLRP3 inflammasome to induce inflammation. It can also cause mitochondrial damage and activate mitophagy. This process mainly relies on the delayed accumulation of the autophagy receptor p62/SQSTM1. The host can maintain its own homeostasis through the ‘NF-κB-p62-mitophagy pathway’ [113]. AMBRA1 acts as a PINK1/PARKIN-independent mitophagy receptor. It is activated by IKKα-mediated phosphorylation at the S1014 site, and mediates the clearance of damaged mitochondria by enhancing its binding to mATG8 family proteins (LC3/GABARAP). HUWE1 can regulate the phosphorylation and activation of AMBRA1, and together with IKKα, forms a HUWE1-IKKα-AMBRA1 synergistic mitophagy pathway, which participates in the molecular network of mitochondrial quality control [114]. TANK binding kinase 1 (TBK1) phosphorylates OPTN to expand its Ub chain binding spectrum and enhance binding stability. It achieves its own recruitment and activation to damaged mitochondria depending on OPTN, forming a signal amplification loop that synergistically regulates both PARKIN-dependent and -independent mitophagy [115]. In addition, mitochondrial ubiquitination and the OPTN-ATG9A axis can autonomously activate mitophagy in a PINK1/PARKIN-independent manner [116].

Regulatory factors also exist in the Rab9-dependent non-classical mitophagy pathway. For example, the transcription factor TFE3 regulates Rab9 transcription by binding to the Rab9 promoter, and Rab9 can be phosphorylated by ULK1 to activate the alternative mitophagy pathway [100]. E2 mediates the upregulation of SIRT1 through estrogen receptors and activates the downstream LKB1-AMPK-ULK1 signal, forming the (SIRT1/LKB1/AMPK/ULK1) pathway. This pathway induces Rab9-dependent non-classical mitophagy to clear damaged mitochondria and maintain mitochondrial function [117].

AMP-activated protein kinase (AMPK) serves as a key regulator of energy metabolism and mitochondrial homeostasis in eukaryotic cells, ensuring cellular energy equilibrium through the orchestration of mitophagy and mitochondrial biogenesis [118]. AMPK modulates the autophagic process of healthy mitochondria based on their functional status, while selectively boosting the mitophagy of dysfunctional mitochondria to eradicate impaired organelles, thereby safeguarding both mitochondrial quantity and quality [119]. In models of Parkinson’s disease, augmentation of the AMPK/mTOR signaling pathway has been shown to enhance mitophagy and ameliorate mitochondrial functional deficiencies, underscoring its potential as a therapeutic target [120].

**Figure 2.**
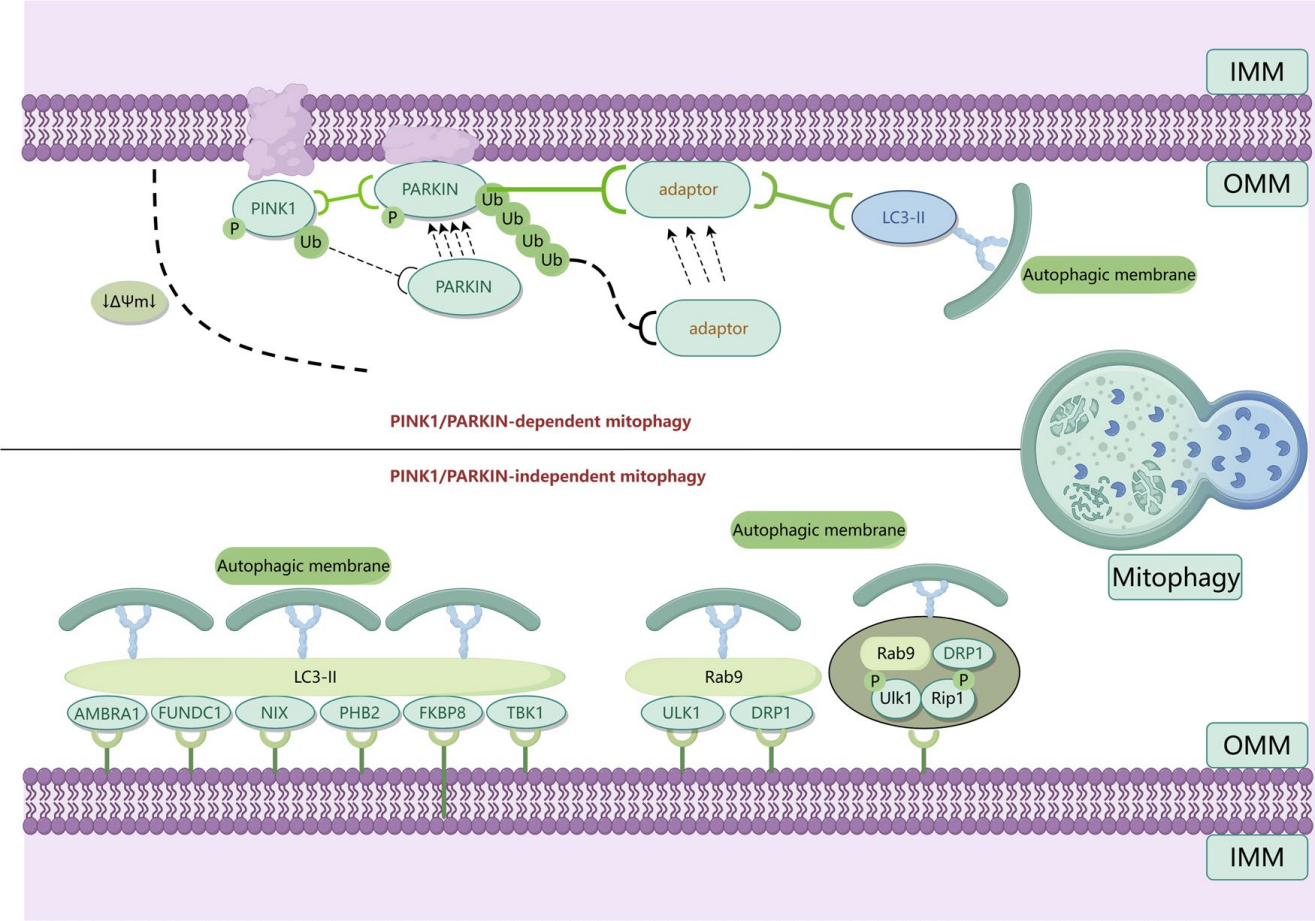
Multiple pathway mechanisms of mitophagy. This figure illustrates two regulatory pathways of mitophagy. The upper part represents the PINK1/PARKIN-dependent pathway: when the mitochondrial membrane potential decreases (ΔΨm↓), PINK1 accumulates on the outer mitochondrial membrane (OMM) and phosphorylates PARKIN. Subsequently, PARKIN mediates the ubiquitination of mitochondrial proteins, recruits LC3-II via adaptor proteins, and drives the autophagic membrane to wrap around mitochondria. The lower part shows the PINK1/PARKIN-independent pathway, which mediates mitophagy through two modes: one is via receptors (e.g. FUNDC1, NIX, AMBRA1) that directly bind to LC3 on the autophagic membrane, and the other is the Rab9-mediated non-canonical mitophagy pathway that is independent of LC3. These two pathways collectively reflect the diversity and complexity of mitophagy regulation. Arrows (→) indicate promotion, and T-bars (⊣) indicate inhibition (the descriptions of the following figures are consistent with this). Note: **IMM**: Inner mitochondrial membrane; **OMM**: Outer mitochondrial membrane; **PINK1**: PTEN induced kinase 1; **PARKIN:** Parkin E3 ubiquitin protein ligase; **Ub**: Ubiquitin; **ΔΨm**: Mitochondrial membrane potential; **LC3-II**: Microtubule-associated proteins light chain 3-II; **AMBRA1**: Autophagy and beclin 1 regulator 1; **FUNDC1**: FUN14 domain containing 1; **NIX**: BNIP3-like protein; **PHB2**: Prohibitin 2; **FKBP8**: FK506 binding protein 8;**TBK1**: TANK binding kinase 1; **Rab9**: Ras-related protein Rab-9; **ULK1**: Unc-51 like autophagy activating kinase 1; **DRP1**: Dynamin-related protein 1;**Rip1**: Receptor-interacting serine/threonine-protein kinase 1.

The aforementioned studies highlight the intricate nature of the regulatory network governing mitophagy, the mechanisms of which are illustrated in Figure 2 offering insights for researchers in future investigations and potential avenues for pharmaceutical interventions.

### 3.4. Dynamic monitoring and detection strategies for mitophagic flux

Mitophagy is a dynamic biological process that cannot be fully evaluated using a single indicator. Mitophagic flux reflects this continuous dynamic process by indicating the overall efficiency of the mitophagic pathway, and serves as a key indicator for assessing mitophagic activity. Transmission electron microscopy represents a classic method for detecting morphological changes in mitophagy and is the gold standard for identifying autophagic structures [121,122]. Nevertheless, the dynamic monitoring of mitophagic flux requires validation by complementary assays.Similar to conventional autophagy detection, mitophagy-related markers (including LC3-II, P62/SQSTM1, PINK1/PARKIN, etc.) are static molecular indicators whose expression levels only reflect a specific step of mitophagy.Specifically, transmission electron microscopy visualizes the formation of mitophagosomes, LC3-II indicates autophagosome formation, P62/SQSTM1 reflects autophagic degradation, and PINK1/PARKIN can be detected as a specific marker of mitophagy.Due to their inability to cover the complete autophagic pathway, the expression levels of these static markers do not fully match the actual activity of the entire autophagic process.Therefore, in mitophagy-related studies, researchers should not only detect the expression of static markers but also perform dynamic tracking and combine multiple auxiliary detection methods to comprehensively and accurately evaluate mitophagic flux [123–127]. The specific methods are as follows: 1) Label mitochondria with mitochondrial markers (e.g. TOM20, VDAC1, COX IV), and perform co-localization detection with autophagy markers or detect their degradation efficiency, which can directly reflect the selective clearance of mitochondria. 2) Detect core autophagy markers(LC3-II/LC3-I, P62/SQSTM1), which can identify autophagosome formation and autophagic flux status. Combined detection with mitochondrial markers can elucidate the specificity of mitophagy and avoid the interference of non-selective autophagy. 3) Detect specific markers of the mitophagy pathway (e.g. PINK1/PARKIN, FUNDC1), which can identify different subtypes of mitophagy (e.g. PINK1/PARKIN-dependent, hypoxia-induced). The activation status of the pathway can be evaluated by fluorescence co-localization, dynamic imaging, or protein modification detection. 4) Combine mitochondrial function indicators (mitochondrial membrane potential ΔΨm, mROS production, ATP synthesis capacity, mtDNA stability, etc.), which can confirm the impact of abnormal mitophagic flux on mitochondrial function.Furthermore, treatment with autolysosome inhibitors (bafilomycin A1 and chloroquine) allows the assessment of autophagic flux by detecting LC3-II accumulation and impaired degradation of mitochondrial proteins, thereby improving the accuracy of mitophagy evaluation [123]. This approach faithfully reflects the dynamic process of mitophagy and avoids research bias caused by single static indicators.Dynamic visualization of autophagy in living cells can be achieved using fluorescence labeling techniques. Fluorescently labeled LC3, combined with markers of autophagosomes and lysosomes, allows real-time tracking of the entire autophagic process. Furthermore, labeling mitochondria, autophagosomes, and lysosomes with fluorescent probes or fluorescent protein fusions, together with multiple imaging and analytical methods, can further advance the visualization of mitophagy [124,128,129].

### 3.5. Detection methods and limitations of mitophagy in renal tissue

Accurate detection of mitophagy is critical for elucidating its regulatory mechanisms in Cis-AKI, as precise assessment of mitophagic activity is essential to clarifying its role in the initiation, progression, and resolution of renal damage. Renal tissue, characterized by high cellular heterogeneity (including RTECs, glomerular cells, and interstitial cells) and inherent vulnerability to ischemia and hypoxia in *ex vivo* settings, poses unique challenges to the reliable detection of mitophagy. Thus, mitophagy detection in renal tissue must be tailored to its unique structural and functional characteristics, and advanced technological approaches should be adopted to overcome the inherent limitations of conventional detection methods, thereby improving the accuracy and specificity of mitophagy assessment in this specialized tissue.

The classic detection methods for mitophagy in renal tissue mainly include the following categories [130]: Transmission electron microscopy enables direct visualization of mitophagosome-mitochondrion complexes but is labor-intensive and cannot distinguish differences in renal substructures. Molecular marker detection (co-localization of LC3 with mitochondrial markers, LC3-II/LC3-I ratio, p62 degradation, etc.) is simple and commonly used, but lacks specificity and may mask the actual mitophagic state of RTECs. Mitophagic flux detection dynamically reflects mitochondrial degradation but is limited by difficult sample processing and low transfection efficiency *in vivo*. Functional detection (mitochondrial membrane potential, ATP, mtROS, etc.) is rapid but shows poor specificity and cannot be used alone to evaluate mitophagy.

In summary, all detection methods for mitophagy in renal tissue have inherent limitations, and their application needs to be optimized and adapted in combination with the characteristics of renal tissue. The above analysis of the detection methods and limitations of mitophagy in renal tissue is not only a tissue-specific supplement to universal detection strategies, but also provides key technical considerations and research boundaries for the subsequent exploration of the interactions between mitophagy and mechanisms such as oxidative stress, inflammation, and PCD in Cis-AKI.

## 4. The interaction between mitophagy and other mechanisms

### 4.1. Oxidative stress and mitophagy

As previously analyzed, all detection methods for mitophagy in renal tissue have inherent limitations, and their application must be optimized according to the unique characteristics of renal tissue. Beyond the technical considerations of mitophagy detection, the regulatory crosstalk between mitophagy and other pathological mechanisms-particularly oxidative stress-is crucial for elucidating the pathogenesis of Cis-AKI and other related diseases. Oxidative stress originates from an imbalance where the production of mtROS exceeds the cellular scavenging capacity, thereby causing damage to biomolecules. Notably, mtROS leakage from mitochondria and impaired mitochondrial repair mechanisms are key drivers of oxidative stress [131]. During disease progression, an intricate regulatory network is formed between oxidative stress and mitophagy, in which the two processes exhibit a mutual regulatory relationship involving both inhibition and promotion; the disruption of this delicate balance often underlies the onset and progression of diseases.

As described above, the PINK1/PARKIN pathway is essential for maintaining mitochondrial function.Disruption of the PINK1/PARKIN pathway hinders the cell’s ability to eliminate oxidatively damaged mitochondria, resulting in the accumulation of mtROS [132]. The ensuing mtROS buildup exacerbates mitochondrial damage, creating a detrimental cycle of ‘mtROS-mitochondrial damage-more mtROS’ [133]. This feedback loop is implicated in various pathological conditions. For instance, high doses of folic acid can trigger mitochondrial dysfunction and mtROS overproduction, leading to excessive mitophagy activation and renal impairment [134]. Oxidative stress in cochlear hair cells triggers mitophagy while inhibiting mitochondrial biogenesis, culminating in mitochondrial damage. The imbalance between mitophagy and mitochondrial biogenesis exacerbates cellular injury under oxidative stress [135]. In ischemic stroke, oxidative stress is a key driver of neuronal damage. Enhancing PINK1/PARKIN-dependent mitophagy in neurons can mitigate mitochondrial mtROS release, ameliorate mitochondrial dysfunction, and reverse oxidative stress-induced neuronal injury in ischemic stroke [136].

Studies have demonstrated that moderate regulation of mitophagy can effectively ameliorate the oxidative stress status. In an acute lung injury (ALI) rat model, inhibiting mitophagy has been shown to mitigate oxidative stress, inflammatory responses, and cellular apoptosis [137]. Using a yeast model, the deletion of mitophagy-related genes results in elevated levels of mtROS and the buildup of dysfunctional mitochondria, underscoring the significance of mitophagy in modulating oxidative stress and preserving mitochondrial function [138]. Within a physiological range, a modest increase in mtROS levels can induce protective mitophagy to maintain cellular homeostasis [139]. The Fanconi anemia complementation group C protein (FANCC) triggers mitophagy by interacting with PARKIN; this interaction restores mitochondrial function, reduces mtROS production, and preserves organelle homeostasis through the coordinated balance between mitophagy and mitochondrial function [140]. These discoveries highlight the regulatory function of the equilibrium between mitophagy and oxidative stress in maintaining cellular functions’ homeostasis.

At the molecular level, researchers have identified key proteins involved in the regulatory network of mitophagy and oxidative stress. Deletion of MFN2 in cardiac tissue impairs PARKIN-dependent mitophagy, leading to the accumulation of damaged mitochondria and subsequent heart failure [141]. Thyroid hormone T3 has been shown to stimulate mtROS generation, activate the mtROS-AMPK signaling pathway, and subsequently phosphorylate the ULK1 protein to initiate mitophagy [142]. Studies in Hela cells have demonstrated that the translocation of ULK1 to mitochondria can elevate superoxide levels, triggering mitophagy; however, excessive activation can reduce ATP production and induce apoptosis [143]. These findings not only elucidate the molecular mechanisms underlying the interplay between oxidative stress and mitophagy but also offer potential therapeutic targets for related diseases.

The above analysis comprehensively elaborates on the regulatory mechanisms, molecular basis, and pathological implications of the interplay between oxidative stress and mitophagy, further supplementing the research context of mitophagy in renal tissue and related diseases. Building on this, subsequent studies can focus on translating these molecular mechanisms into clinical practice, exploring targeted regulatory strategies for the mitophagy-oxidative stress axis, and providing more effective therapeutic ideas for the prevention and treatment of Cis-AKI and other related diseases.

### 4.2. Inflammation and mitophagy

The inflammatory response is a crucial defense mechanism that the body employs to combat infections or injuries. It involves the activation of immune sensors upon detecting danger signals, the release of pro-inflammatory mediators, and the recruitment of immune cells to eliminate threats and initiate the repair process [144]. This intricate process relies on a complex signaling network, primarily encompassing the NF-κB pathway, NLRP3 inflammasome pathway, the TNF signaling pathway, and the Janus Kinase-Signal Transducer and Activator of Transcription pathway **(**JAK-STAT pathway), which intricately interact through precise molecular regulation [145]. Despite their distinct mechanisms of action, both the inflammatory response and mitophagy share the common objective of upholding cellular and tissue homeostasis, underscoring a closely intertwined regulatory relationship between them.

The NF-κB transcription factor family plays a pivotal role in regulating inflammation, immunity, and cell survival, with its aberrant activation being closely linked to the pathogenesis of various diseases [146]. Research indicates that disrupted mitophagy results in the accumulation of dysfunctional mitochondria, leading to heightened mtROS production. These mtROS, serving as crucial second messengers, can trigger the NF-κB signaling pathway *via* oxidative modifications [147]. Consequently, NF-κB activation can stimulate the NLRP3 inflammasome, prompting the release of inflammatory mediators and causing mitochondrial impairment. Nonetheless, NF-κB also possesses the ability to suppress NLRP3 inflammasome activation by upregulating p62, thereby promoting PARKIN-dependent mitophagy to eliminate impaired mitochondria and mitigate excessive inflammation [113]. This intricate interplay suggests that NF-κB and mitophagy contribute to maintaining tissue homeostasis and facilitating tissue repair through a bidirectional regulatory mechanism.

In contrast-induced kidney injury, enhancing PINK1/PARKIN-dependent mitophagy reduces mtROS production and inhibits NLRP3 inflammasome activation, thereby exerting a renoprotective effect [59]. The protective role of this pathway has also been verified in neuropathy models [148,149]^.^ Stimulator of interferon genes (STING) is a key regulatory molecule in cytoplasmic DNA-induced type I interferon responses and can trigger neuroinflammation in Parkinson’s disease. Upregulation of classical PINK1/PARKIN-dependent mitophagy, however, can alleviate STING-mediated inflammatory responses [150]. In virus-induced inflammation, activating BNIP3 receptor-mediated mitophagy decreases mtROS production and inhibits NLRP3 inflammasome activation [151]. Similarly, inducing ULK1-dependent mitophagy suppresses NLRP3 inflammasome activation, reduces excessive inflammatory responses, and thus protects damaged cells [152]^.^ Additionally, increased expression of the mitophagy receptor FUNDC1 activates mitophagy, which improves chronic inflammation by inhibiting NLRP3 inflammasome activation and reducing the release of inflammatory mediators [153]. The inner mitochondrial membrane protein PHB2 can directly initiate mitophagy as a receptor and also regulate PINK1/PARKIN pathway-mediated mitophagy. Following enhanced mitophagy through this dual role, PHB2 inhibits NLRP3 inflammasome activation and protects RTECs from damage [154,155]. In muscle tissue from patients with inclusion body myositis (IBM), abnormal mitophagy induced by enhanced oxidative stress and mitochondrial dysfunction in the inflammatory microenvironment leads to abnormal NLRP3 inflammasome activation and disrupts cellular homeostasis [156]. Furthermore, BNIP3 deficiency reduces mitophagy and exacerbates cellular damage; inhibiting the NLRP3 inflammasome pathway promotes the interaction between hypoxia-inducible factor 1α (HIF1A) and BNIP3, thereby upregulating BNIP3-mediated mitophagy and ultimately alleviating mitochondrial damage and exerting a protective effect [157]. These findings suggest that enhancing mitophagy through multiple pathways is an effective strategy to inhibit the inflammatory cascade. These mechanisms reveal the flexible and tissue-specific interaction between mitophagy and inflammatory pathways, highlighting the context dependence of mitophagy in regulating inflammatory responses.

The role of mitophagy may be tissue-specific. Recent studies have shown that PARKIN can exacerbate airway inflammation by promoting the release of mitochondrial DNA (mtDNA) in airway tissue [158]. Excessive mtDNA release can trigger a strong TLR9/MyD88/NF-κB-mediated inflammatory response [159]. Estrogen receptor β (ESR2) can upregulate PINK1 expression in lung tissue by transcriptionally inhibiting miR-423, which targets PINK1 mRNA, thereby exacerbating asthma [160]. These findings are contrary to those previously reported in kidney or neural tissue.The authors speculate that this difference is most likely due to the tissue-specific immune microenvironment. In the kidney, the low density of immune cells limits mtDNA release. In the airway immune microenvironment, however, immune cells are abundant. Under pathological conditions, this may lead to rapid recruitment of immune cells, resulting in immune dysregulation and excessive mitophagy.

In lipid metabolism research, the absence of PINK1 inhibits mitophagy, causes mitochondrial functional impairments, and upregulates NLRP3 expression. Interestingly, these alterations do not trigger the conventional inflammatory pathway but instead promote adipocyte differentiation [161]. This finding implies a non-canonical regulatory association between PINK1 and NLRP3 in adipocyte differentiation, highlighting their regulatory roles in cells *via* the ‘metabolism-differentiation axis’ rather than the conventional ‘mitochondria-inflammation axis.’

**Figure 3.**
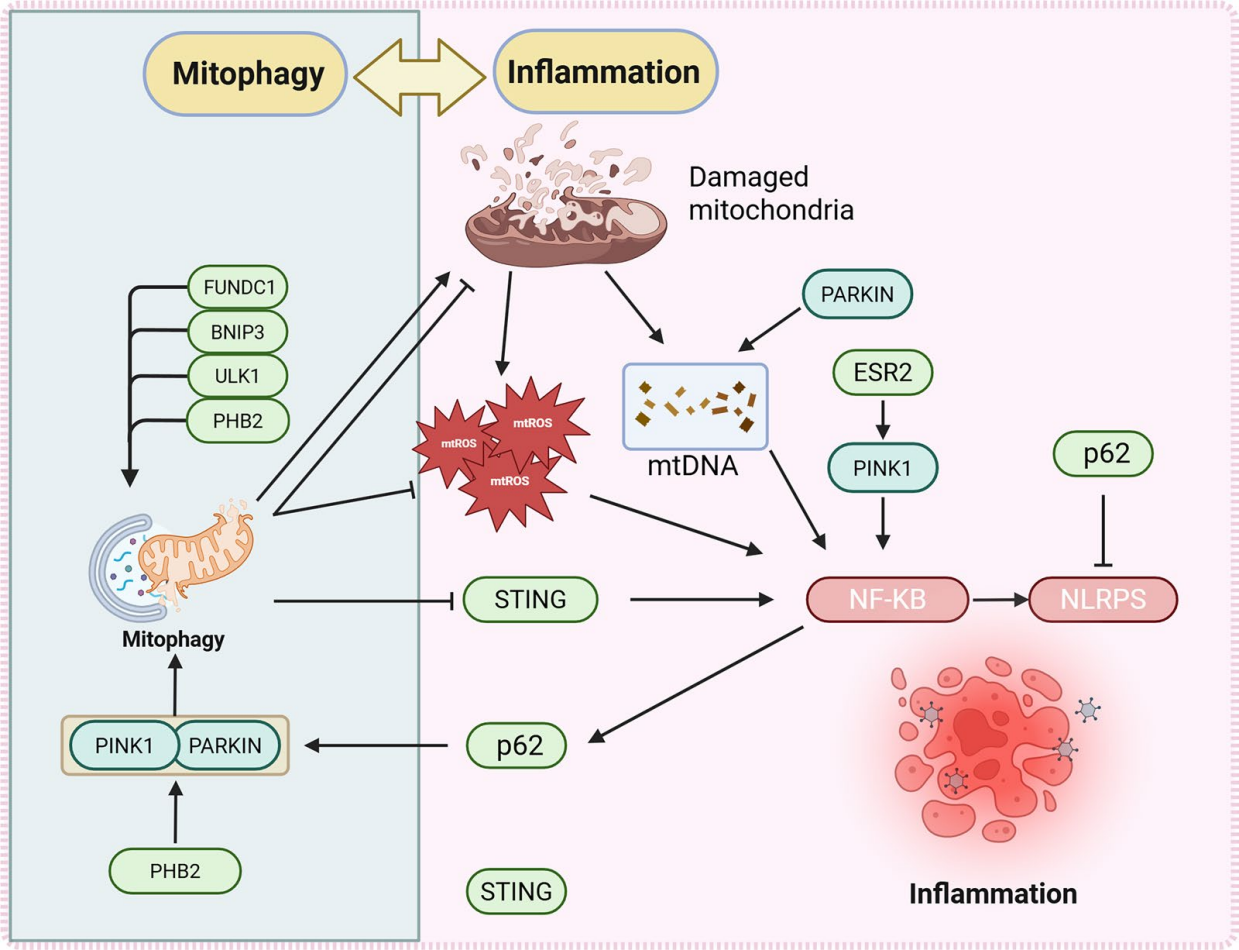
Mitophagy regulates inflammation. This figure illustrates the regulatory relationship between mitophagy and inflammatory responses. Mitophagy can clear mtROS and damaged mitochondria, reduce the release of mtDNA, thereby inhibiting the downstream NF-κB-NLRP3 inflammasome pathway and suppressing inflammatory responses. In addition, inflammation can feedback-regulate mitophagy, forming a bidirectional regulatory network between mitophagy and inflammation. Note: **FUNDC1**: FUN14 domain containing 1; **BNIP3**: BCL2/adenovirus E1B 19 kDa interacting protein 3; **ULK1**: Unc-51 like autophagy activating kinase 1; **PHB2**: Prohibitin 2; **PINK1**: PTEN induced kinase 1; **PARKIN**: Parkin RBR E3 ubiquitin protein ligase; **STING**: Stimulator of interferon genes; **mtROS**: Mitochondrial reactive oxygen species; **mtDNA**: Mitochondrial DNA; **NF-κB**: Nuclear Factor-kappa B; **NLRP3**: NOD-like receptor family pyrin domain containing 3; **p62**: Sequestosome 1; **ESR2**: Estrogen receptor 2.

In essence, dysfunctional mitochondria can undergo clearance *via* mitophagy facilitated by either PINK1/PARKIN-dependent pathways or receptor-dependent pathways. This process diminishes the generation of mtROS and the release of mtDNA, consequently impeding the initiation of inflammatory cascades. Such constitutes a fundamental mechanism governing the interplay between mitophagy and inflammation. Collectively, these discoveries delineate an intricate regulatory framework governing the reciprocal modulation of mitophagy and inflammatory reactions (Figure 3), offering a novel vantage point for comprehending the etiology of associated disorders.

### 4.3. Apoptosis and mitophagy

Cellular homeostasis relies on the coordinated regulation of PCD and organelle quality control. Apoptosis and mitophagy serve as pivotal mechanisms that preserve tissue homeostasis and mediate stress responses. Apoptosis, a fundamental form of PCD, is executed through intrinsic (mitochondrial-dependent) and extrinsic (death receptor-dependent) pathways [162,163]. Apoptosis and mitophagy engage in a sophisticated and tightly interconnected interplay.

The BCL-2 protein family is classified into three groups based on its regulatory functions in apoptosis: anti-apoptotic proteins (BCL-2, BCL-xL, BCL-w, MCL-1), pro-apoptotic proteins (BAX, BAK), and BH3-only proteins (PUMA, NOXA, BNIP3/BNIP3L) [164]. This family exhibits tight crosstalk with PINK1/PARKIN-dependent mitophagy, jointly maintaining cellular homeostasis.Parkin preserves cellular and tissue homeostasis *via* a dual mechanism: ‘mild damage—mitophagic repair’ and ‘severe damage—apoptotic elimination’. The key mechanism underlying Parkin-mediated apoptosis involves PINK1-dependent ubiquitination and degradation of Myeloid cell leukemia 1 (Mcl-1). Upon mild mitochondrial injury, Parkin preferentially triggers mitophagy to remove damaged mitochondria. When mitochondria undergo severe depolarization, PARKIN cooperates with PINK1 to promote ubiquitination and proteasomal degradation of the anti-apoptotic protein Mcl-1, thereby relieving inhibition of Bax/Bak and initiating apoptosis to eliminate severely damaged cells [165]. The BCL-2 family participates in mitochondrial quality control by regulating the PARKIN/PINK1-dependent mitophagy pathway. The core mechanisms are as follows: anti-apoptotic Bcl-2 family members (Bcl-xL, Mcl-1, Bcl-W, etc.) suppress mitophagy by blocking Parkin translocation to depolarized mitochondria. In contrast, BH3-only proteins (PUMA, NOXA, etc.) neutralize these anti-apoptotic proteins and thus reverse the inhibition of mitophagy [105]. As a key anti-apoptotic factor, Bcl-2 inhibits apoptosis and prolongs cell survival, partly through interaction with c-Myc [166,167]. Under physiological conditions, cytosolic Parkin catalyzes monoubiquitination of Bcl-2 to enhance its protein stability. Stabilized Bcl-2 then binds to Beclin 1 to restrain excessive autophagy, ensuring cellular homeostasis. Upon mitochondrial damage, Parkin translocates to mitochondria and switches function to promote mitophagy for the clearance of impaired organelles [168]. BCL-xL is another critical anti-apoptotic protein with multifaceted regulatory roles. On one hand, BCL-xL can be phosphorylated by PINK1 and exerts anti-apoptotic effects independently of PARKIN [169]. On the other hand, BCL-xL forms oligomers with PARKIN to hinder its translocation from the cytosol to mitochondria. Meanwhile, BCL-xL interacts with mitochondrial PINK1 and suppresses PINK1-mediated PARKIN mitochondrial targeting. Together, these two mechanisms inhibit mitophagy [170]. Pro-apoptotic proteins Bax/Bak also modulate mitophagy. They not only induce mitochondrial injury and apoptosis but also enhance mitophagic activity by promoting LC3-II localization to mitochondria [171,172]. Furthermore, Bax/Bak are directly inhibited by Parkin, which limits excessive apoptosis, facilitates efficient removal of damaged mitochondria, and attenuates potential pro-inflammatory effects. PUMA is a canonical BH3-only protein that primarily mediates pro-apoptotic signaling and serves as a key component of the p53-dependent apoptotic pathway [173]. PUMA interacts closely with BCL-xL and regulates autophagy in a bidirectional and localization-dependent manner. In the cytoplasm, PUMA inhibits autophagy. After translocating to mitochondria, PUMA forms a PUMA-BCL-xL-ULK1 complex to promote mitophagy [174]. BNIP3, another vital BH3-only protein, coordinately regulates apoptosis and mitophagy. It suppresses pro-survival Bcl-2 family members *via* the BH3 domain to promote apoptosis, and initiates mitophagy by binding to autophagy-related proteins through the LIR motif [175]. Bcl-2-L13, a unique member of the BCL-2 family, displays tissue-specific functions and cannot be simply categorized as pro- or anti-apoptotic [176]. Both *in vivo* and *in vitro* studies demonstrate that Bcl-2-L13 induces mitochondrial fragmentation and elevates mitophagic flux [177,178].

Several well-established mitophagy-related molecules also play a role in regulating apoptosis. BNIP3, a key mitophagy regulator, not only triggers mitophagy but also enhances the permeability of the OMM to facilitate apoptosis [175]. In the context of myocardial ischemia-reperfusion injury, ischemic conditions can trigger mitophagy by dephosphorylating the FUNDC1 protein. Mitophagy prevents apoptosis by engulfing mitochondrial fragments and Cyt C [179].

AMPK acts as an energy sensor, coordinating mitophagy and apoptosis. Exposure to arsenic can disrupt these two processes (mitophagy and apoptosis) in poultry hepatocytes by inhibiting the AMPK signaling pathway [180]. The tumor suppressor p53 plays a crucial role in regulating mitophagy and apoptosis. In a fatty liver model, p53 induces mitophagy *via* Damage-Regulated Autophagy Modulator (DRAM), upregulates Bax to enhance apoptosis, and modulates cAMP/PKA pathway-mediated apoptosis by increasing PUMA/NOXA levels [181,182]. The ERK signaling pathway serves as a key regulator at the intersection of mitophagy and apoptosis, enhancing apoptosis and mitigating kidney injury by balancing mitophagy [183].

**Figure 4.**
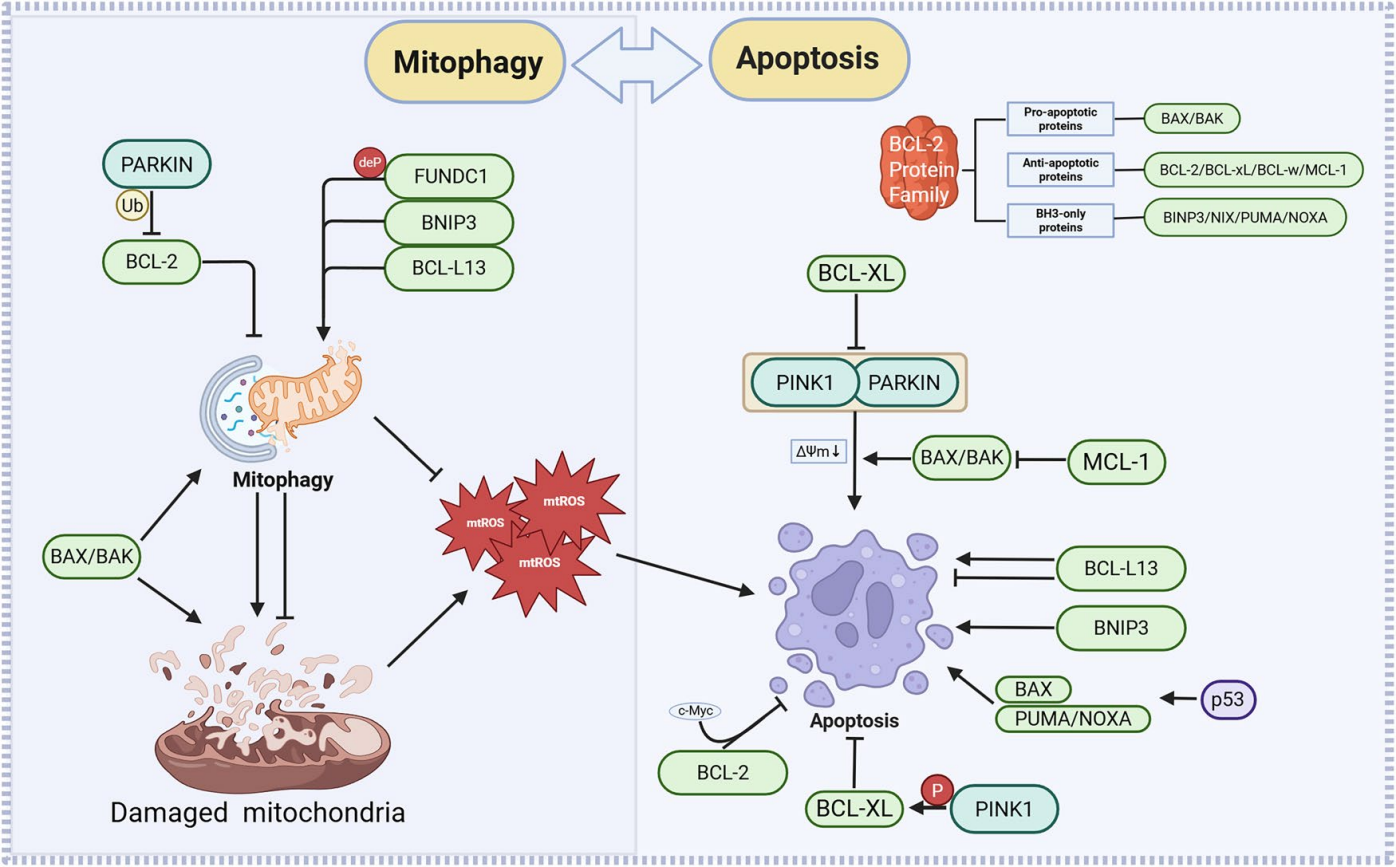
Crosstalk between mitophagy and apoptosis. This schematic illustrates the core regulatory network connecting mitophagy and apoptosis in response to mitochondrial damage. Mitophagy is mediated by PARKIN‑dependent ubiquitination and receptor‑mediated pathways (e.g. FUNDC1, BNIP3, BCL‑L13, DEAM), which are regulated by BCL‑2 family proteins. Apoptosis is controlled by BCL‑2 family proteins. MtROS acts as a central hub, coordinating the balance between mitophagy‑mediated cell survival and apoptosis during mitochondrial quality control. Note: **PARKIN**: Parkin E3 ubiquitin protein ligase; **FUNDC1**: FUN14 domain containing 1; **BNIP3**: BCL2/adenovirus E1B 19 kDa interacting protein 3; **BCL-L13**: BCL2-like 13; **PINK1**: PTEN induced kinase 1; **ΔΨm**: Mitochondrial membrane potential; **BCL-2**: B-cell lymphoma 2; **BCL-XL**: B-cell lymphoma-extra large; **MCL-1:** Myeloid cell leukemia 1; **BCL-w**: B-cell lymphoma 2 family proteins; **BAX**: BCL2-associated X protein; **BAK**: BCL2 antagonist/killer 1; **BH3-only proteins**: BCL2 homology 3-only proteins; **NIX**: BNIP3-like protein; **PUMA**: p53 upregulated modulator of apoptosis; **NOXA**: Phorbol-12-myristate-13-acetate-induced protein 1; **p53**: Tumor protein p53; **c-Myc**: Myc proto-oncogene protein; **mtROS**: Mitochondrial reactive oxygen species.

The aforementioned studies suggest a nuanced molecular crosstalk between mitophagy and apoptosis, with associated molecules potentially assuming varied functions in distinct pathophysiological contexts (Figure 4).

### 4.4. Pyroptosis and mitophagy

Pyroptosis is a PCD process initiated by inflammasome activation. Key characteristics include GSDM family protein-induced cell membrane permeabilization and the release of pro-inflammatory mediators. Upon sensing danger signals like infections, metabolic challenges, or tissue injury, the NLRP3 inflammasome is triggered. This leads to Gasdermin D (GSDMD) cleavage, facilitating the production and secretion of inflammatory cytokines such as IL-1β and IL-18, culminating in pyroptosis [184–186]. Notably, mitochondrial dysfunction and consequent mtROS accumulation play pivotal roles in inflammasome activation [187]. Mitophagy, a quality control mechanism, selectively eliminates impaired mitochondria to uphold mitochondrial homeostasis, thus underscoring mitochondrial impairment as a central node in the regulatory circuits of pyroptosis and mitophagy.

At the molecular level, mitophagy, facilitated by various pathways, regulates pyroptosis. The PINK1/PARKIN pathway enhances mitophagy, ameliorates mitochondrial dysfunction, and eliminates mtROS. PARKIN also directly ubiquitinates NLRP3, thereby inhibiting inflammasome activation, reducing caspase-1 and IL-1β protein expression, and suppressing GSDMD-mediated pyroptosis [188,189]. Another pathway involving BNIP3/NIX regulates pyroptosis by modulating mitophagy. Decreased BNIP3 levels impede mitophagy, leading to mtROS accumulation, NLRP3 inflammasome activation, and subsequent promotion of pyroptosis [190]. Additionally, the mitochondrial metabolic regulator VDAC1 influences NLRP3 inflammasome activation in retinal capillary endothelial cells, thereby modulating pyroptosis and mitophagy [191,192]. Activation of the AMPK-mTOR-TFEB signaling pathway induces mitophagy, reduces mtROS levels, diminishes Caspase-1, GSDMD, and NLRP3 expression, and inhibits pyroptosis [193]. The deacetylase Sirtuin 3 (SIRT3) enhances mitophagy by deacetylating PINK1, which in turn inhibits NLRP3 inflammasome assembly, consequently impeding pyroptosis [194,195]. Collectively, these findings underscore the protective role of mitophagy in inhibiting inflammasome activation and pyroptosis by mitigating mtROS generation.

The N-terminal fragment of Gasdermin D (GSDMD-NT) disrupts both the IMM and OMM, resulting in mitochondrial damage, release of inflammatory mediators, inflammasome activation, and subsequent pyroptosis. Mitophagy serves a protective role by restoring mitochondrial functional homeostasis [196]. Given that mitophagy exerts a protective effect against pyroptosis, targeting mitophagy to enhance pyroptosis has become a potential strategy for tumor therapy. For example, incorporating chloroquine, a mitophagy inhibitor, into biomimetic nanoparticles can impede mitophagy’s protective effect, enhance tumor cell pyroptosis, induce tumor cell death, and exert its anti-tumor efficacy [197]. Inhibition of PINK1 can elevate mtROS levels and impede mitophagy. Additionally, pyroptosis is triggered through the caspases-Gasdermin E (GSDME) pathway, leading to tumor cell death [198,199]. These findings highlight potential targets for modulating mitophagy and pyroptosis. Tumor progression can be inhibited by suppressing mitophagy and enhancing pyroptosis in tumor cells, offering novel avenues for refining tumor treatment strategies.

**Figure 5.**
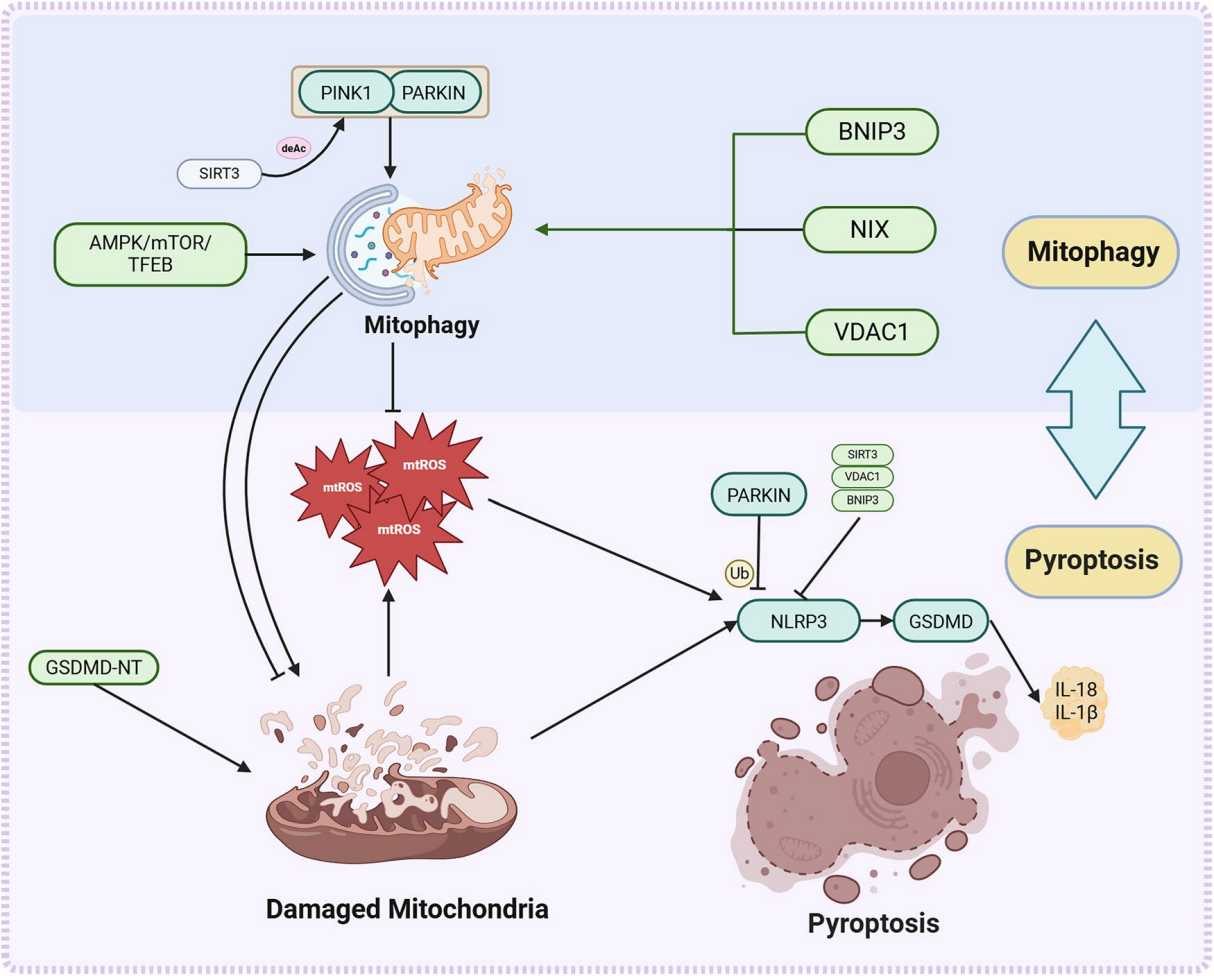
Regulatory network of mitophagy in modulating pyroptotic. This schematic illustrates the regulatory network linking mitophagy and pyroptosis upon mitochondrial damage. Mitophagy (PINK1/PARKIN, BNIP3/NIX/VDAC1, AMPK/mTOR/TFEB) clears damaged mitochondria and limits mtROS. Excess mtROS activates the NLRP3 inflammasome, triggering GSDMD-mediated pyroptosis and inflammatory cytokine release, forming a feedback loop with mitochondrial injury. Note: **PINK1**: PTEN induced kinase 1; **PARKIN**: Parkin E3 ubiquitin protein ligase; **SIRT3**: Sirtuin 3; **AMPK**: AMP-activated protein kinase; **mTOR**: mechanistic target of rapamycin; **TFEB**: transcription factor EB; **BNIP3**: BCL2/adenovirus E1B 19 kDa interacting protein 3; **NIX**: BNIP3-like protein; **VDAC1**: Voltage-dependent anion channel 1; **Ub**: Ubiquitin; **deAc**: Deacetylation; **NLRP3**: NOD-like receptor family pyrin domain containing 3; **GSDMD:** Gasdermin D**; GSDMD-NT**: Gasdermin D N-terminal domain; **IL-1β**: Interleukin-1β; **IL-18**: interleukin-18; **mtROS**: Mitochondrial reactive oxygen species.

These studies have elucidated the reciprocal regulation between pyroptosis and mitophagy, establishing a dynamic equilibrium crucial for cell fate determination (Figure 5). Precise modulation of the mitophagy-pyroptosis balance holds promise for innovative therapeutic interventions across diverse pathologies.

### 4.5. Ferroptosis and mitophagy

Ferroptosis is an iron-dependent form of PCD distinct from apoptosis, pyroptosis, and necrosis. Its initiation and progression are not mediated by classical apoptosis-associated proteases or inflammasomes, but characterized by three core biochemical features: excessive iron ion accumulation, irreversible intracellular lipid peroxide buildup, and impaired function of the antioxidant defense system centered on GPX4. Concurrently, it is accompanied by characteristic morphological and subcellular structural alterations. The most distinctive early feature is the atrophy and loss of mitochondrial cristae, along with other specific morphological changes including increased mitochondrial membrane density and reduced mitochondrial volume [200,201]^.^ As the central regulatory site for intracellular iron metabolism and a key locus of lipid peroxidation, mitochondria play an irreplaceable core role in the initiation and progression of ferroptosis. On the one hand, mitochondria participate in the storage, transport, and utilization of cellular iron, and their internal iron pool serves as an important source of intracellular labile iron. Excessive iron can induce massive ROS production *via* the Fenton reaction, directly triggering lipid peroxidation in both mitochondria and the cytoplasm. On the other hand, mitochondrial membranes are rich in polyunsaturated fatty acids, making them susceptible targets of lipid peroxidation. Furthermore, the endogenous antioxidant system of mitochondria is vulnerable to iron overload and oxidative stress. Once the level of lipid peroxidation exceeds the cellular scavenging capacity, mitochondrial membrane damage and functional collapse will be induced, ultimately driving the full onset of ferroptosis [202,203].

Mitophagy maintains cellular homeostasis by selectively clearing dysfunctional mitochondria, thereby reducing intracellular peroxide accumulation and serving as a crucial negative regulatory mechanism of ferroptosis. In rectal cancer organoid models, mitophagy effectively inhibits the production of lipid peroxides and the accumulation of mtROS, significantly decreasing cellular susceptibility to ferroptosis. In contrast, tumor cells with impaired mitochondrial function exhibit a marked increase in ferroptosis sensitivity due to the loss of this protective mechanism [204]. Mitophagy mediated by distinct signaling pathways exerts specific regulatory effects on ferroptosis. FUNDC1-mediated mitophagy interacts with acyl-CoA synthetase long-chain family member 4 (ACSL4) to alleviate ferroptosis and confer protective effects against injury in cardiomyocytes [205]. Conversely, FUNDC1 can bind to GPX4 and promote its translocation to mitochondria, where GPX4 is degraded during mitophagy, ultimately triggering cellular ferroptosis and exacerbating cell injury [206]. As core regulators of mitophagy, BNIP3/NIX deficiency leads to massive mtROS accumulation and mitochondrial dysfunction, with only partial antioxidant stress counteraction achieved *via* the activation of the nuclear factor E2-related factor 2 (NRF2) pathway. Precise modulation of BNIP3/NIX-mediated mitophagy not only helps maintain cellular redox balance and eliminate dysfunctional mitochondria that generate ROS, thereby inhibiting ferroptosis [207], but also drives the P62-KEAP1-NRF2 signaling pathway to preserve cellular iron and redox homeostasis, thus suppressing ferroptosis [208]. Targeted activation of the NRF2 pathway enhances PARKIN-dependent mitophagy and upregulates GPX4 expression, thereby inhibiting ferroptosis and delaying disease progression [209]. AMPK phosphorylates PINK1 to potentiate PINK1/PARKIN pathway-mediated mitophagy, which reduces excessive mtROS release and consequently exerts a synchronous inhibitory effect on oxidative stress, inflammation and ferroptosis [210,211]. In addition, targeted modulation of SIRT3 can also upregulate mitophagy levels, effectively inhibit ferroptosis and restore normal mitochondrial function [212]. Collectively, these findings confirm that mitophagy attenuates the progression of ferroptosis through multiple pathways and targets, exerting important cytoprotective effects.

While mitophagy predominantly inhibits ferroptosis under homeostatic or mild stress conditions, its role shifts under specific pathological scenarios – instead suppressing ferroptosis, mitophagy can contribute to its induction. For example, in ionizing radiation-induced cellular damage, the clearance of damaged mitochondria *via* mitophagy results in the release of large amounts of free fatty acids and labile iron from mitochondria, which exacerbates lipid peroxidation and ultimately induces ferroptosis [213]. Similarly, inhibition of protein O-GlcNAcylation (a post-translational modification that stabilizes DRP1) promotes DRP1-mediated mitochondrial fragmentation, enhances mitophagy, and increases labile iron accumulation,and rendering cells more susceptible to ferroptosis [214]. In neuronal cells, Protein Kinase C δ (PKCδ)-activated Specificity Protein 1 (SP1)-dependent mitophagy triggers a decline in mitochondrial membrane potential and mtROS buildup, thereby fostering ferroptosis [215]. The interaction between SIRT3 and BNIP3 inhibits aberrant mitophagy and ferroptosis mediated by the BNIP3/NIX pathway, thereby averting the onset and progression of steroid-induced osteonecrosis of the femoral head (SIONFH) [212]. Furthermore, oxidative stress-induced activation of the Cyclic GMP-AMP Synthase-Stimulator of Interferon Genes Pathway (cGAS-STING) can also induce ferroptosis by enhancing mitophagy [216]. These findings collectively indicate that mitophagy exerts a context-dependent regulatory influence on ferroptosis.

**Figure 6.**
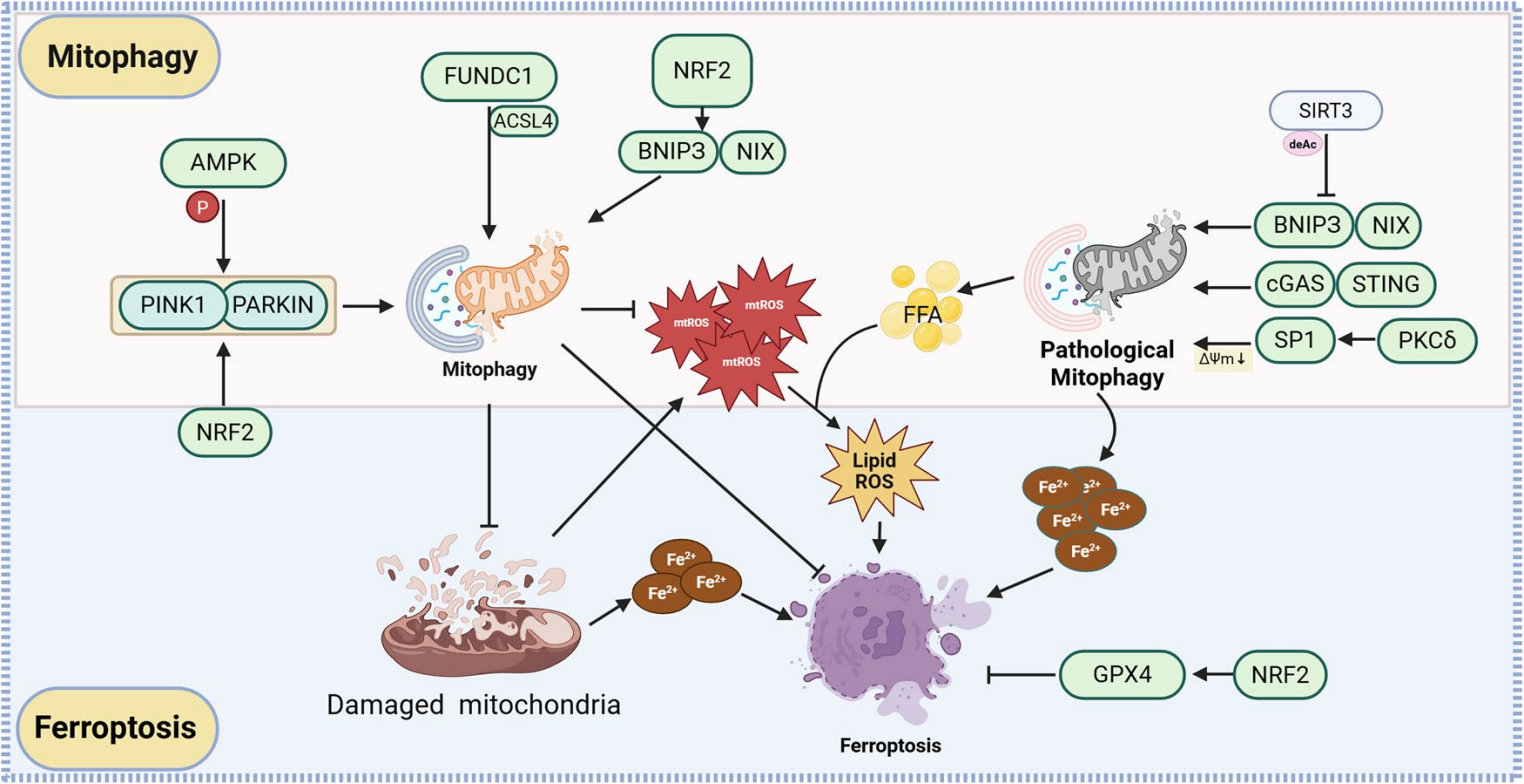
The regulatory relationship between mitophagy and ferroptosis. This schematic illustrates the regulatory network linking mitophagy and ferroptosis in response to mitochondrial damage. Canonical mitophagy (PINK1/PARKIN, and receptor pathways including FUNDC1/ACSL4, BNIP3/NIX) clears damaged mitochondria to limit mtROS, lipid ROS, and iron overload, thereby suppressing ferroptosis. Under partial regulatory conditions, pathological mitophagy (mediated by SIRT3, BNIP3/NIX, cGAS/STING, and PKCδ/SP1) exacerbates mitochondrial dysfunction, promoting ferroptosis through GPX4 inhibition and iron/lipid ROS accumulation. Note: **PINK1**: PTEN induced kinase 1;PARKIN: Parkin E3 ubiquitin protein ligase; **AMPK**: AMP-activated protein kinase; **NRF2**: Nuclear factor erythroid 2-related factor 2; **FUNDC1**: FUN14 domain containing 1; **ACSL4**: Acyl-CoA synthetase long-chain family member 4; **BNIP3**: BCL2/adenovirus E1B 19 kDa interacting protein 3;**NIX**: BNIP3-like protein; **SIRT3**: Sirtuin 3; cGAS: Cyclic GMP-AMP synthase; **STING**: Stimulator of interferon genes; **SP1**: Specificity protein 1; **PKCδ**: Protein kinase C delta; **ΔΨm**: Mitochondrial membrane potential; **deAc**: Deacetylation; **GPX4**: Glutathione peroxidase 4; **FFA**: Free fatty acid; **Lipid ROS**: Lipid reactive oxygen species; **mtROS**: Mitochondrial reactive oxygen species

From these seemingly contradictory findings, it can be concluded that cells exhibit distinct adaptive regulatory mechanisms under different pathological conditions. Under homeostatic conditions, mitophagy exerts a cytoprotective effect primarily by clearing damaged mitochondria, thus sustaining cellular survival. However, under intense stress or upon activation of specific signaling pathways, excessive mitophagy may lead to the abnormal accumulation of metabolites (Figure 6), which in turn promotes ferroptosis. Collectively, these findings demonstrate that mitophagy exerts a bidirectional regulatory effect on ferroptosis, and the specific regulatory factors remain to be elucidated, meriting further in-depth investigation by researchers.

### 4.6. Interactions of multiple mechanisms in Cis-AKI

Previous sections have clarified mitophagy’s crosstalk with oxidative stress, inflammation, and various PCD pathways. Cis-AKI arises not from isolated pathways but from the synergy of multiple pathological mechanisms centered on mitochondrial dysfunction. Mitophagy acts as a key regulatory node for all these pathways. This section integrates these mechanisms to clarify their synergistic modes and mitophagy’s central regulatory role.

**Figure 7.**
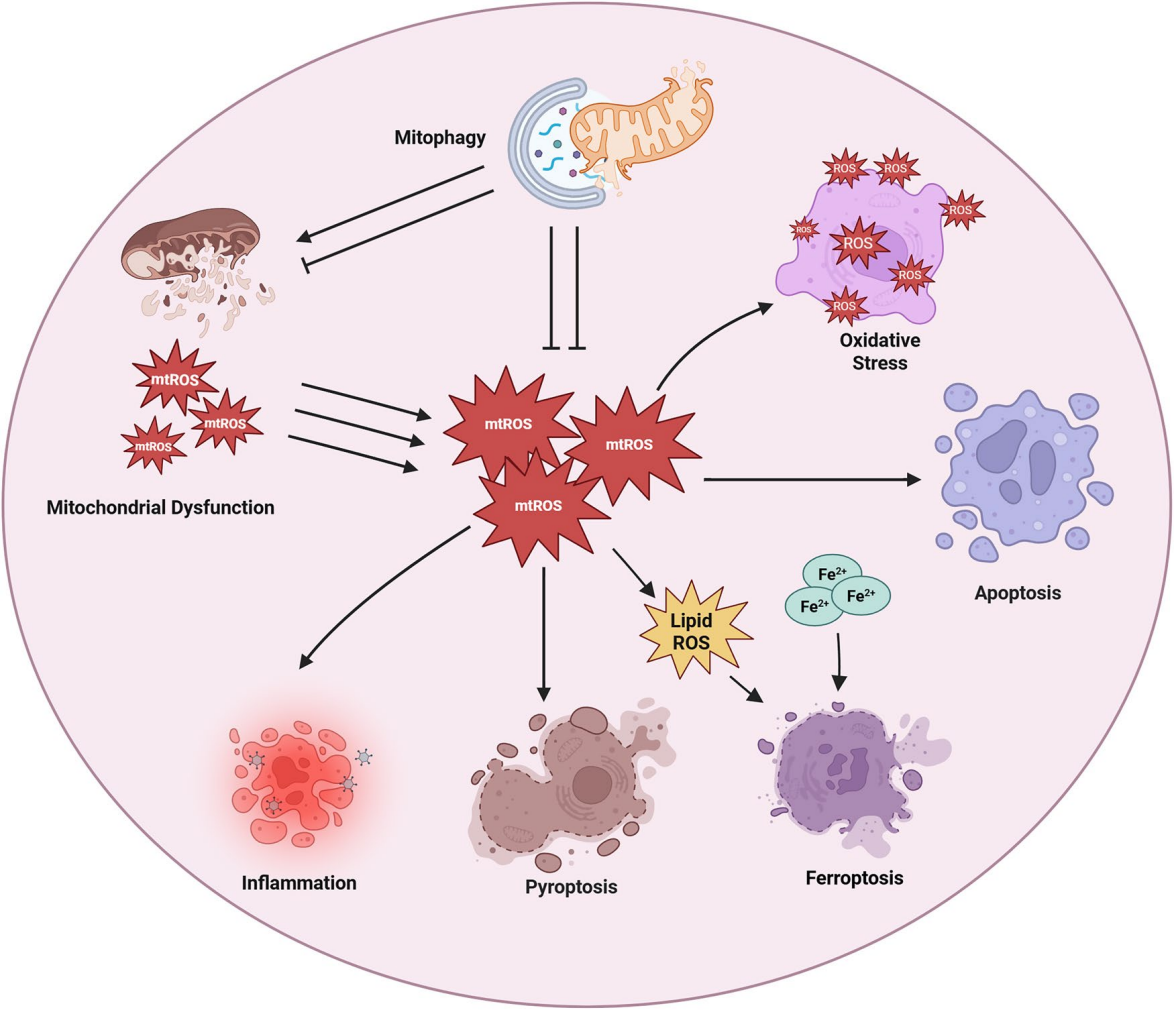
Mitochondrial ROS as a central hub coordinating mitophagy and multiple cell death pathways. This schematic illustrates the regulatory network centered on mitochondrial ROS (mtROS) in mitochondrial quality control. Mitochondrial dysfunction drives mtROS accumulation, which reciprocally modulates mitophagy to clear damaged organelles. Excess mtROS triggers oxidative stress and downstream cell death programs (apoptosis, pyroptosis, ferroptosis) alongside inflammatory responses, highlighting mtROS as a core mediator linking mitochondrial homeostasis to cell fate decisions. Note: **mtROS**: Mitochondrial reactive oxygen species; **Lipid ROS**: Lipid reactive oxygen species; **ROS**: Reactive oxygen species

Cis-AKI is mediated through various mechanisms, including the classical apoptotic pathway involving Fas and TNFR1: two members of the TNF receptor family [217]. Mitochondrial damage is closely linked to cisplatin nephrotoxicity, as it disrupts mitochondrial membrane permeability, leading to Cyt C release and activation of the caspase-9-dependent apoptotic pathway in a dose- and time-dependent manner [218,219]. Beyond apoptosis, cisplatin also upregulates TNF-α-mediated inflammation, exacerbating kidney injury by increasing pro-inflammatory cytokines and chemokines [220]. Oxidative stress also contributes significantly, with cisplatin-induced mtROS generation damaging mitochondria and causing lipid peroxidation, worsening nephrotoxicity [25,221]. Recent studies have identified additional cell death pathways involved in Cis-AKI. Pyroptosis, a lytic cell death triggered by inflammasome activation and mediated by caspase-1/11, has been implicated [222]. Although early research suggested that caspase-1 is inactive in cisplatin-treated RTECs, subsequent studies clarified that downregulating caspase-1 alleviated Cis-AKI – providing direct evidence for pyroptosis involvement [223,224]. Further investigations demonstrated that cisplatin upregulates caspase-11 expression, leading to GSDMD cleavage, pore formation on cell membranes, and pyroptotic renal tubular injury [225]. More recently, the caspase-3/GSDME pathway – where caspase-3 cleaves GSDME to switch from apoptotic to pyroptotic signaling – can also mediate pyroptosis, contributing to kidney injury [226]. Furthermore, ferroptosis, characterized by lipid peroxidation, is implicated in cisplatin nephrotoxicity, as evidenced by significantly increased lipid peroxide levels (a hallmark of ferroptosis) in kidney tissue following cisplatin treatment [227]. The activation of ferroptosis-related genes through the Farnesoid X receptor (FXR) effectively mitigates Cis-AKI, underscoring the involvement of ferroptosis [228]. Collectively, Cis-AKI is driven by an interconnected network of mechanisms, with mtROS acting as a central hub that links mitochondrial dysfunction to multiple injury pathways: mtROS disrupts mitochondrial membrane permeability to activate the caspase-9-dependent apoptotic pathway [219], activates the NLRP3 inflammasome (*via* mtDNA release) to induce pyroptosis [225], and promotes lipid peroxidation (by inhibiting GPX4 activity) to trigger ferroptosis [227]. Targeting mtROS or its downstream effectors – for instance, *via* mitophagy (which clears mtROS-producing damaged mitochondria) – may therefore simultaneously mitigate multiple injury pathways (Figure 7).

## 5. Regulatory role and research status of mitophagy in Cis-AKI

As previously discussed, the pathogenesis of Cis-AKI, exemplified by agents such as cisplatin, entails intricate interplays among diverse mechanisms and signaling cascades. A pertinent query arises: within this intricate pathological milieu, is there a singular intervention site that could ameliorate multiple injurious mechanisms concurrently by modulating a solitary pathway or molecular entity? Emerging evidence suggests that mtROS may represent the linchpin of this regulatory framework, with mitophagy emerging as a pivotal regulatory nexus.

Upon cellular stimulation, changes in mitochondrial membrane potential lead to mtROS accumulation, facilitating the stabilization of PINK1 on the OMM. PINK1 phosphorylates PARKIN, activating its ubiquitin ligase activity and initiating damaged mitochondria clearance [229,230]. MtROS accumulation not only activates the NF-κB signaling pathway and NLRP3 inflammasome – triggering inflammatory responses – but also induces cellular apoptosis and pyroptosis [231,232]^.^ Meanwhile, the excessive accumulation of mtROS caused by mitochondrial dysfunction can trigger lipid peroxidation, which is a typical characteristic of ferroptosis [233]. Cis-AKI injury involves excessive mitochondrial damage, abnormal mtROS release, and subsequent ferroptosis. Subsequent activation of mitophagy mitigates oxidative stress, inflammation, and ferroptosis *via* the mtROS/HO-1/GPX4 axis – by enhancing GPX4-mediated lipid peroxide clearance – thereby exerting renoprotective effects [234,235]. Mitophagy modulates cell inflammation by clearing damaged mitochondria through PARKIN-dependent mitophagy, reducing the TLR4/NF-κB/NLRP3 inflammatory response. Notably, this protective effect is context-dependent; excessive mitophagy, however, may release mtDNA – subsequently activating the NF-κB pathway and exacerbating inflammation [159,236]. This dual effect of mitophagy may reflect compensatory activation or tissue-specific regulatory mechanisms. NLRP3 inflammasome activation increases inflammatory factor release, leading to gasdermin D-dependent pyroptosis [180,186]. Mitochondria are pivotal in cell damage, with mitochondrial dysfunction linking various cell death pathways (apoptosis, pyroptosis, ferroptosis). Modulating mitophagy, enhancing mitochondrial function, and reducing mtROS production are therefore key strategies for ameliorating Cis-AKI. Consistent with this mechanism, translational studies have demonstrated that targeting mitophagy *via* pharmacological agents can protect against Cis-AKI.

In translational medicine, studies have shown that targeting mitophagy with certain drugs can protect against Cis-AKI. For instance, the antioxidant α-mangostin (αM) mitigates oxidative stress, preserves mitochondrial proteins, and enhances mitophagy to reduce cell damage [233–237]. Farrerol, in a chronic kidney injury model, activates NRF2-targeted antioxidant enzymes (HO-1 and NQO1) and induces PINK1/PARKIN-dependent mitophagy by recruiting LC3-II and the p62/SQSTM1 receptor protein, thereby reducing chronic inflammation and fibrosis [238]. Targeting the transcription factor EB (TFEB) can mitigate cisplatin-induced mitochondrial dysfunction by promoting mitophagy, thus offering renal protection [239]. Small molecules – such as the PINK1 activator kinetin riboside and the mTOR inhibitor rapamycin – enhance mitophagy through the PINK1/PARKIN pathway, thereby reducing Cis-AKI [38,240]. Novel nanomaterials – including ultra-small tungsten-based nanodots (TWND) and COPT (a cisplatin-targeted nanomaterial) – promote mitophagy and protect renal function by scavenging mtROS and targeting specific renal sites [241,242]. AMPK agonists (e.g. C24 and metformin) activate mitophagy by modulating PINK1 localization and activity, thereby reducing oxidative stress and tubular injury [243,244]. The mitochondria-targeted antioxidant MitoQ protects the kidneys through the NRF2/PINK1 pathway [245]. Interventions like remote ischemic preconditioning (rIPC) and tannic acid-cerium nanozymes can ameliorate mitochondrial dysfunction and ferroptosis, alleviating kidney injury. Collectively, these findings highlight that modulating mitophagy – *via* diverse molecular mechanisms ranging from pharmacological activation to nanomaterial targeting – serves as a crucial ‘multi-target hub’ for therapeutic intervention in Cis-AKI [246]^.^ Such innovative strategies not only provide novel mechanistic insights but also lay a foundation for developing clinically translatable approaches to prevent and treat chemotherapy-related renal damage.

## 6. Prospects: the dual regulatory role and therapeutic potential of mitophagy in Cis-AKI

Cis-AKI remains a major clinical dilemma that restricts anticancer efficacy and worsens patient prognosis. As a core mitochondrial quality control mechanism, mitophagy has emerged as a pivotal dual-regulatory node in this pathological process, exerting context-dependent protective or detrimental effects. This review summarizes the regulatory role of mitophagy in Cis-AKI and outlines promising therapeutic prospects to guide future translational research.

### 6.1. Quantitative distinction between protective and pathological mitophagy in Cis-AKI

Moderate mitophagy exerts renoprotective effects by selectively eliminating damaged mitochondria, the primary source of mtROS. This process not only reduces mtROS accumulation to alleviate oxidative stress but also inhibits downstream inflammatory cascades (e.g. NF-κB/NLRP3 pathway) and modulates PCD pathways including apoptosis, pyroptosis, and ferroptosis. Preclinical studies have validated that activating mitophagy *via* pharmacological agents (e.g. PINK1 agonists, AMPK modulators), natural compounds (e.g. α-mangostin, farrerol), or nanomaterial-based strategies effectively mitigates cisplatin-induced tubular injury. Conversely, excessive mitophagy disrupts mitochondrial homeostasis in high-energy-demand renal tubules, depletes functional mitochondria, and promotes mtDNA release to exacerbate inflammation and cell death – underscoring its context-dependent dual regulatory role in Cis-AKI.

Given the complex regulatory properties of mitophagy, there is currently no unified criterion for defining its dual protective and deleterious effects in a context-dependent manner. Based on the core theories of mitophagy summarized above, the early distinction of its functional tendency can be explored from the following dimensions:First, the rate and integrity of autophagic flux serve as the core criteria for evaluation. By treating cells with autophagolysosomal inhibitors such as bafilomycin A1 and chloroquine, combined with double labeling of mitochondrial markers (TOM20/VDAC1) and the autophagic marker (LC3-II), the status of autophagic flux can be accurately evaluated. If LC3-II accumulates significantly and the degradation of mitochondrial markers is impaired after inhibitor treatment, while live-cell imaging shows that the entire process of ‘mitochondrial damage – autophagosome encapsulation – lysosomal degradation’ is short in duration with rapid recovery of mitochondrial function, this indicates unobstructed autophagic flux, which is a protective effect. In contrast, if LC3-II does not accumulate significantly but mitochondrial markers continue to increase, accompanied by prolonged process duration and mitochondrial function collapse, this suggests blocked autophagic flux, which is an injurious effect.Second, functional orientation is clarified by combining the specificity of autophagic subtypes. Increased PINK1/PARKIN phosphorylation levels, a high colocalization rate with mitochondria, and decreased P62 expression indicate the selective clearance of damaged mitochondria, which is a protective effect. Activation of the PINK1/PARKIN pathway with sustained elevation of P62 suggests failure of selective clearance. Elevated pan-autophagic markers (e.g. LC3-II, Beclin1) and non-selective colocalization between mitochondria and autophagosomes indicate non-specific injury, which is prone to disrupting cellular homeostasis.Third, the judgment is strengthened through the linkage verification of ‘autophagic flux indicators – mitochondrial function – cell fate’. Unobstructed autophagic flux is often accompanied by improved mitochondrial function (mROS, ΔΨm, ATP) and no elevation of apoptotic markers, consistent with the characteristics of a protective effect. Abnormal autophagic flux is mostly manifested by deteriorated mitochondrial function and increased apoptotic/necrotic markers, indicating an injurious effect.Finally, spatiotemporal specificity is used for auxiliary confirmation. Temporally, in the early stage of Cis-AKI, activation of the PINK1/PARKIN pathway and decreased P62 are mostly protective effects; in the late stage, accumulation of LC3-II and mitochondrial markers is mostly injurious. Spatially, minimal expression of autophagic markers restricted to the injured area (coinciding with the functional repair area) suggests a protective effect. In contrast, overexpression in normal areas or massive accumulation in injured areas indicates an injurious effect.

In summary, mitophagy exerts a context-dependent dual regulatory role in Cis-AKI. Moderate activation confers renoprotection by selectively clearing damaged mitochondria, mitigating oxidative stress, suppressing inflammatory cascades, and modulating PCD pathways. In contrast, excessive mitophagy disrupts mitochondrial homeostasis in renal tubules (which have high energy demands) and exacerbates kidney injury. Currently, there is no unified criterion for defining these dual protective and deleterious effects. Nevertheless, the functional tendency of mitophagy can be differentiated through four key dimensions: autophagic flux integrity, autophagic subtype specificity, the linkage between mitochondrial function and cell fate, and spatiotemporal specificity. This framework not only lays a theoretical foundation for further investigating the precise regulatory mechanisms of mitophagy and optimizing clinical translation strategies but also provides robust theoretical support for the clinical application of mitophagy-targeted therapies.

### 6.2. Clinical translation bottlenecks of mitophagy-targeted therapy for Cis-AKI

Although mitophagy-targeted therapy has shown significant renoprotective potential in basic research on Cis-AKI, no relevant regimens have entered clinical research to date. Its clinical translation is hindered by four core bottlenecks that act as key obstacles to practical application:First, human research evidence is severely lacking, creating an inherent gap between basic and clinical research. Most studies are limited to *in vitro* RTECs and mouse models, with scarce clinical data on Cis-AKI in cancer patients. Moreover, animal models typically involve single-factor-induced injury in healthy individuals, which differs markedly from the complex pathophysiology of clinical cancer patients, who often receive concurrent chemoradiotherapy and have underlying organ dysfunction. As a result, basic research findings cannot be directly translated to clinical practice.Second, drug delivery lacks tissue specificity, increasing the risk of systemic off-target effects. All existing regulatory agents are systemic formulations that fail to achieve precise delivery to renal tissue, particularly RTECs. Systemic regulation not only induces abnormal mitophagy in non-target tissues such as the heart, liver and tumors, causing additional tissue damage, but also reduces the effective drug concentration in the kidneys, thus weakening the renoprotective effect.Third, the interaction mechanism with cisplatin remains unclear, posing a risk of compromised antitumor efficacy. Cisplatin induces apoptosis in tumor cells by damaging mitochondrial function, whereas mitophagy-targeted agents primarily regulate mitochondrial homeostasis. Their interaction pattern remains undefined; blind combination may reverse the antitumor effect of platinum-based drugs and even promote tumor cell survival.Fourth, clinical dose regulation is challenging due to the narrow therapeutic window of mitophagy in Cis-AKI. Mitophagy exerts both protective and injurious effects, yet no clinically applicable methods for quantitative detection of mitophagic activity are available. This leads to potential dose inaccuracies: insufficient activation at low doses, or excessive mitophagy at high doses, resulting in energy metabolic disorders in RTECs and exacerbated kidney injury.

To address these obstacles, targeted research should be conducted to facilitate clinical translation:First, clinically relevant translational research should be performed. Cis-AKI animal models that simulate clinical features of cancer patients should be established, and small-sample, single-center early clinical studies should be carried out to verify the efficacy and safety of mitophagy-targeted regulation in humans, thus bridging basic research and clinical application.Second, kidney- or RTEC-specific delivery systems should be developed. Using nanocarriers and kidney-targeting ligands, precise delivery systems for mitophagy-targeted agents can be constructed to enhance drug enrichment in RTECs, reduce distribution in non-target tissues, and minimize off-target effects.Third, the combined mechanism of mitophagy-targeted agents and platinum-based drugs should be systematically investigated at cellular and animal levels. Regulatory targets or agents that exert synergistic renoprotective and antitumor effects should be screened, and combination regimens should be established to balance renoprotection and chemotherapeutic efficacy.Finally, clinically accessible methods for quantitative mitophagy detection and individualized dose regulation should be developed. Non-invasive biomarkers such as plasma mtDNA release kinetics and urinary LC3-II/p62 ratio can be used to guide clinical dose adjustment, thereby preventing pathological mitophagy caused by excessively high doses. These biomarkers, which reflect mitophagic flux and mitochondrial damage, enable real-time monitoring of mitophagic activity in patients receiving cisplatin chemotherapy, helping clinicians individualize dose regimens to balance renoprotection and antitumor efficacy.

In conclusion, mitophagy represents a promising ‘multi-target hub’ for mitigating Cis-AKI. Addressing current translational barriers through tissue-specific targeting, precise regulation, and advanced mechanistic exploration will pave the way for developing novel therapeutic strategies that improve the quality of life for cancer survivors.

## Data Availability

This is a review article and does not report any original experimental data. Thus, no additional data are associated with this work.
